# Supplementary material for: Profiling IgG and IgA antibody responses during vaccination and infection in a high-risk gonorrhoea population
Source: Nat Commun. 2024 Aug 7;15:6712. doi: 10.1038/s41467-024-51053-x (PMC11306574; doi:10.1038/s41467-024-51053-x)
Supplement: Supplementary file 1 — Supplementary Information [file 41467_2024_51053_MOESM1_ESM.pdf]

Supplementary Information

Contents

Figure S1 Heatmaps of vaccine recipient responses. 2
Figure S2 tSNE separation of Opa antigens. 3
Figure S3 Alignment of sequences of Opa proteins used in the microarray. 4
Figure S4 Example correlations of IgG and IgA responses in the vaccinated cohort for selected Opa protein variants. 5
Figure S5 PCA analysis applied to IgG and IgA immunoprofiles in the vaccinated cohort at the 10 weeks timepoint. 6
Figure S6 PCA analysis applied to IgG and IgA immunoprofiles in the vaccinated cohort at the 24 weeks timepoint. 7
Figure S7 Violin plots of bactericidal activity (% recovery) at three serum dilutions. 8
Figure S8 Violin plots of ELISpot activity. 9
Table S1 Peptides used in ELISpot assays 10
Clinical Trial Protocol 11

Figure S1 Heatmaps of vaccine recipient responses. Antigens are ordered in columns, with the same order used for each block. Participants are numbered in rows (numbering is arbitrary). The viridis colour scheme is used, with lowest signals purple/blue, through to yellow for the highest responses. Arrows indicate columns of highly reacting antigens.

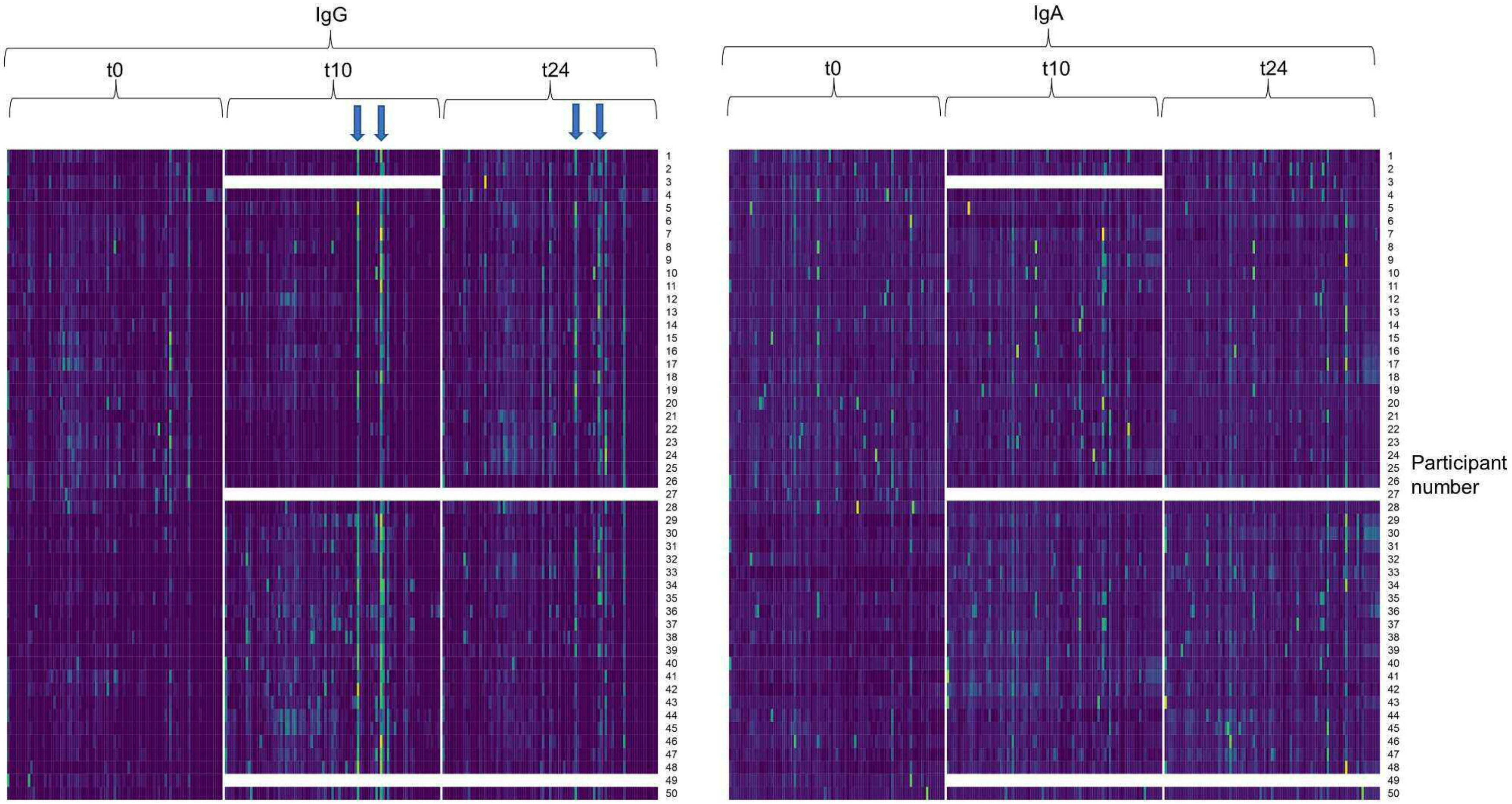

Figure S2 tSNE separation of Opa antigens. The plots use the same data from Fig 3B and C; Opa variants are labelled (sequences in Fig S3). The OpaD/Opa9/Opa58 grouping is circled in red.

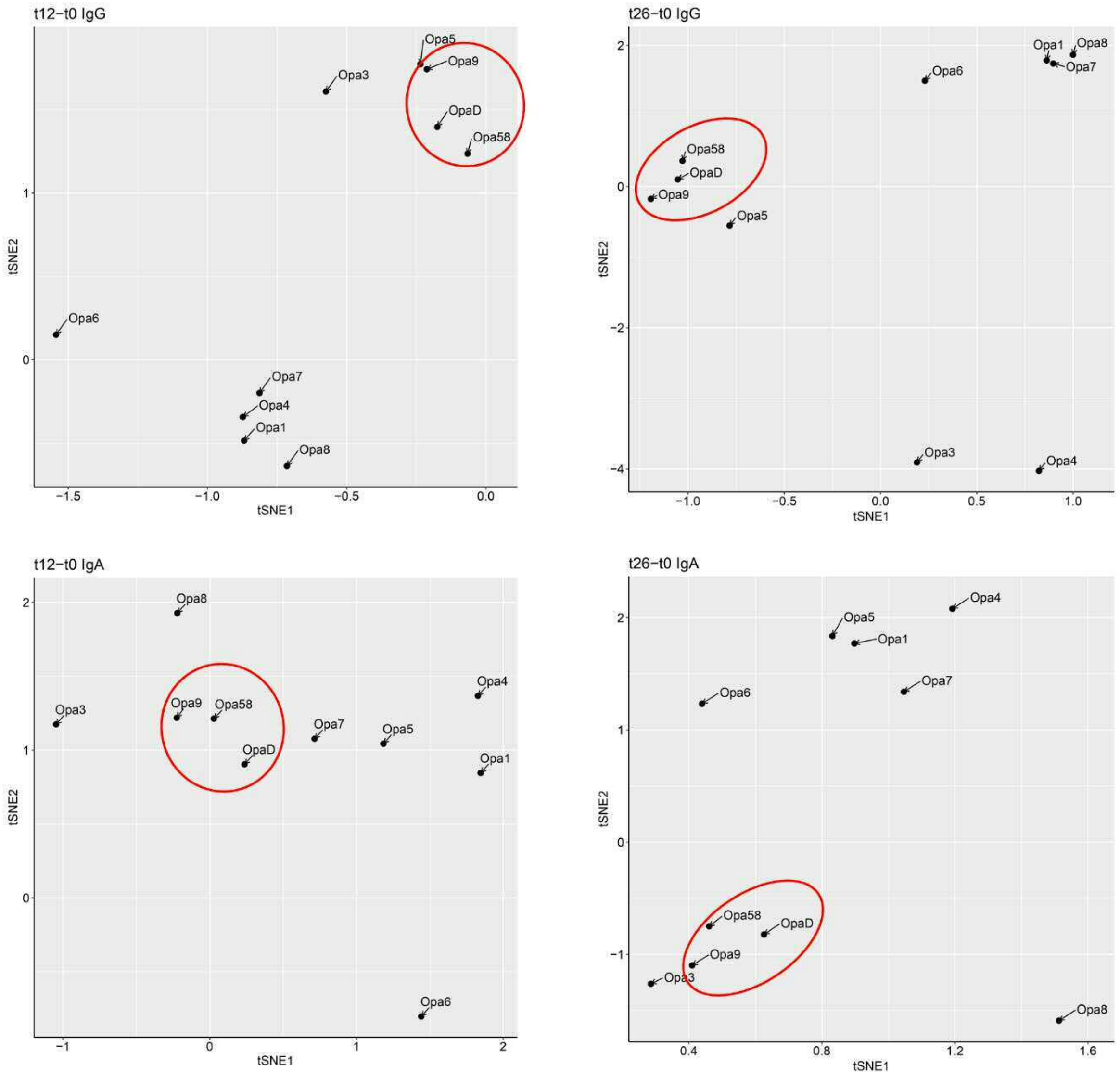

Figure S3 Alignment of sequences of Opa proteins used in the microarray. Alignment was carried out using Clustalx. Of the 11 Opas in the genome two were identical in sequence (Opa2 and Opa7); Opa2 was removed. OpaD/Opa9/Opa58 tend to cluster together in tSNE plots (Fig S2) and are highlighted in yellow. The second hypervariable loop region is highlighted in red for these variants.

```
Opa7        MKETAAAKFERQHMDSPDLGTDDDKAMA----------VQADLAYAAERITHDYPEPTGA     50
Opa1        MKETAAAKFERQHMDSPDLGTDDDKAMA----------VQADLAYAAERITHDYPEPTGA     50
Opa3        MKETAAAKFERQHMDSPDLGTDDDKAMA----------VQADLAYAYEHITRDYPDAAGA     50
Opa4        MKETAAAKFERQHMDSPDLGTDDDKAMA----------VQADLAYAAERITHDYPEPTGT     50
Opa5        MKETAAAKFERQHMDSPDLGTDDDKAMA----------VQADLAYAAERITHDYPEPTGA     50
Opa8        MKETAAAKFERQHMDSPDLGTDDDKAMA----------VQADLAYAYEHITRDYPDAAGA     50
Opa6        MKETAAAKFERQHMDSPDLGTDDDKAMA----------VQADLAYAYEHITRDYPDAAGA     50
OpaD        MKETAAAKFERQHMDSPDLGTDDDKAMAASEGNGRGPYVQADLAYAAERITHDYPEPTAP     60
Opa9        MKETAAAKFERQHMDSPDLGTDDDKAMA----------VQADLAYAAERITHDYPEPTAP     50
Opa58       MKETAAAKFERQHMDSPDLGTDDDKAMAASEGNGRGPYVQADLAYAAERITHDYPEPTAP     60
            ****************************          ******** *:**:***: :.

Opa7        KKDKKISTVSDYFRNIRTHSVHPRVSVGYDFGSWRIAADYARYRKWNNSKYSVNIKRVKE     110
Opa1        KKG-KISTVSDYFRNIRTHSIHPRVSVGYDFGGWRIAADYARYRKWNNSKYSVNTKKVNE     109
Opa3        NQGKKISTVSDYFKNIRTHSIHPRVSVGYDFGGWRIAADYARYRKWNDNKYSVDIKELEN     110
Opa4        KK-DKISTVSDYFRNIRTHSIHPRVSVGYDFGGWRIAADYARYRKWNNSKYSVNTKKVNE     109
Opa5        KKG-KISTVSDYFRNIRTHSIHPRVSVGYDFGGWRIAADYARYRKWNDNKYSVNIKELGR     109
Opa8        NQGKKISTVSDYFKNIRTRSVHPRLAFGYDFGGWRIAADYARYRKWHNNKYSVNIKELGR     110
Opa6        NKG-KISTVSDYFRNIRTHSIHPRVSVGYDFGGWRIAADYARYRKWHNNKYSVNIKELER     109
OpaD        GKN-KISTVSDYFRNIRTHSIHPRVSVGYDFGGWRIAADYARYRKWHNNKYSVNIKELER     119
Opa9        GKN-KISTVSDYFRNIRTHSIHPRVSVGYDFGGWRIAADYARYRKWNDNKYSVDIKELEN     109
Opa58       GKN-KISTVSDYFRNIRTHSIHPRVSVGYDFGGWRIAADYARYRKWNDNKYSVDIKELEN     119
             :  *********:****:*:***::.*****.*************::.****: *.: .

Opa7        NNGSGK----KLTQDLKTENQENGTFHAVSSLGLSAVYDFDTGSRFKPYAGVRVSYGHVR     166
Opa1        NKGEKI----NVTQYLKAENQENGTFHAVSSLGLSAVYDFKLNDKFKPYIGMRVGYGHVR     165
Opa3        KNQN--------KRDLKTENQENGSFHAVSSLGLSAVYDFKLNDKFKPYIGARVAYGHVR     162
Opa4        NKGEKI----NVTQYLKAENQENGTFHAVSSLGLSAVYDFKLNDKFKPYIGARVAYGHVR     165
Opa5        KDGTSSSGRYLNIQTRKTENQENGTFHAVSSLGLSTVYDFRANDKFKPYIGVRVAYGHVR     169
Opa8        NDNSASDSKHLNIKTQKTEHQENGTFHAVSSLGLSTVYDFRANDKFKPYIGVRVAYGHVR     170
Opa6        KNNKTFGGNQLNIKYQKTEHQENGTFHAVSSLGLSTVYDFRVNDKFKPYIGVRVGYGHVR     169
OpaD        KNNKTFGGNQLNIKYQKTEHQENGTFHAVSSLGLSAVYDFKLNDKFKPYIGARVAYGHVR     179
Opa9        KNQN--------KRDLKTENQENGSFHAVSSLGLSAVYDFKLNDKFKPYIGARVAYGHVR     161
Opa58       KNQN--------KRDLKTENQENGSFHAVSSLGLSAVYDFKLNDKFKPYIGARVAYGHVR     171
            :.           :  *:*:****:**********:****  ..:**** * **.*****

Opa7        HSIDSTKKTTDVITAPPTTSDGAPTTYN----ANPQTQNPYHQSDSIRRVGLGVIAGVGF     222
Opa1        HQVRSVEQETTTVTTYL--QSGKPSPIV----RGSTLKLPHHESRSSRRLGFGAMAGVGI     219
Opa3        HSIDSTKKTTEFLTAAG--QDGGAPTVY----NNGSTQDAHQESDSIRRVGLGVIAGIGF     216
Opa4        HSIDSTKKTTEFLTAAG--QDGGAPTVY----NNGSTQDAHQESDSIRRVGLGVIAGVGF     219
Opa5        HQVHSMEKETTTVTTYP--SDGSAK---TSVPSEMPPKPAYHENRSSRRLGFGAMAGVGI     224
Opa8        HQVHSMEKETTTVTTYP--SDGSAK---TSVPSEMPPKPAYHENRSSRRLGFGAMAGVGI     225
Opa6        HGIDSTKKTKNTLTAYH--SAGTKPTYYDDIDSGKNQKNTYRQNRSSRRLGFGAMAGVGI     227
OpaD        HSIDSTKKITGTLTAYP--SDADAAV--TVYPDGHPQKNTYQKSNSSRRLGFGAMAGVGI     235
Opa9        HSIDSTKKITGTLTAYP--SDADAAV--TVYPDGHPQKNTYQKSNSSRRLGFGAMAGVGI     217
Opa58       HSIDSTKKITGTLTAYP--SDADAAV--TVYPDGHPQKNTYQKSNSSRRLGFGAMAGVGI     227
            * : * :: .  :*:    . .               :  :::. * **:*:*.:**:*:

Opa7        DITPNLTLDTGYRYHNWGRLENTRFKTHEASLGMRYRF  260
Opa1        DVAPGLTLDAGYRYHYWGRLENTRFKTHEASLGVRYRF  257
Opa3        DITPKLTLDTGYRYHNWGRLENTRFKTHEASLGVRYRF  254
Opa4        DITPNLTLDAGYRYHNWGRLENTRFKTHEASLGMRYRF  257
Opa5        DVAPGLTLDAGYRYHYWGRLENTRFKTHEASLGMRYRF  262
Opa8        DVAPGLTLDAGYRYHYWGRLENTRFKTHEASLGVRYRF  263
Opa6        DVAPGLTLDAGYRYHYWGRLENTRFKTHEASLGVRYRF  265
OpaD        DVAPGLTLDAGYRYHNWGRLENTRFKTHEASLGMRYRF  273
Opa9        DVAPGLTLDAGYRYHNWGRLENTRFKTHEASLGMRYRF  255
Opa58       DVAPGLTLDAGYRYHNWGRLENTRFKTHEASLGMRYRF  265
            *::* ****:***** ****************:****
```

Figure S4 Example correlations of IgG and IgA responses in the vaccinated cohort for selected Opa protein variants. Raw fluorescence values are plotted for A) IgG b) IgA. Points are coloured as follows: t0, black; t10, blue; t24 red. Left hand panels are for OpaD versus Opa9; right hand panels are for OpaD versus Opa58.

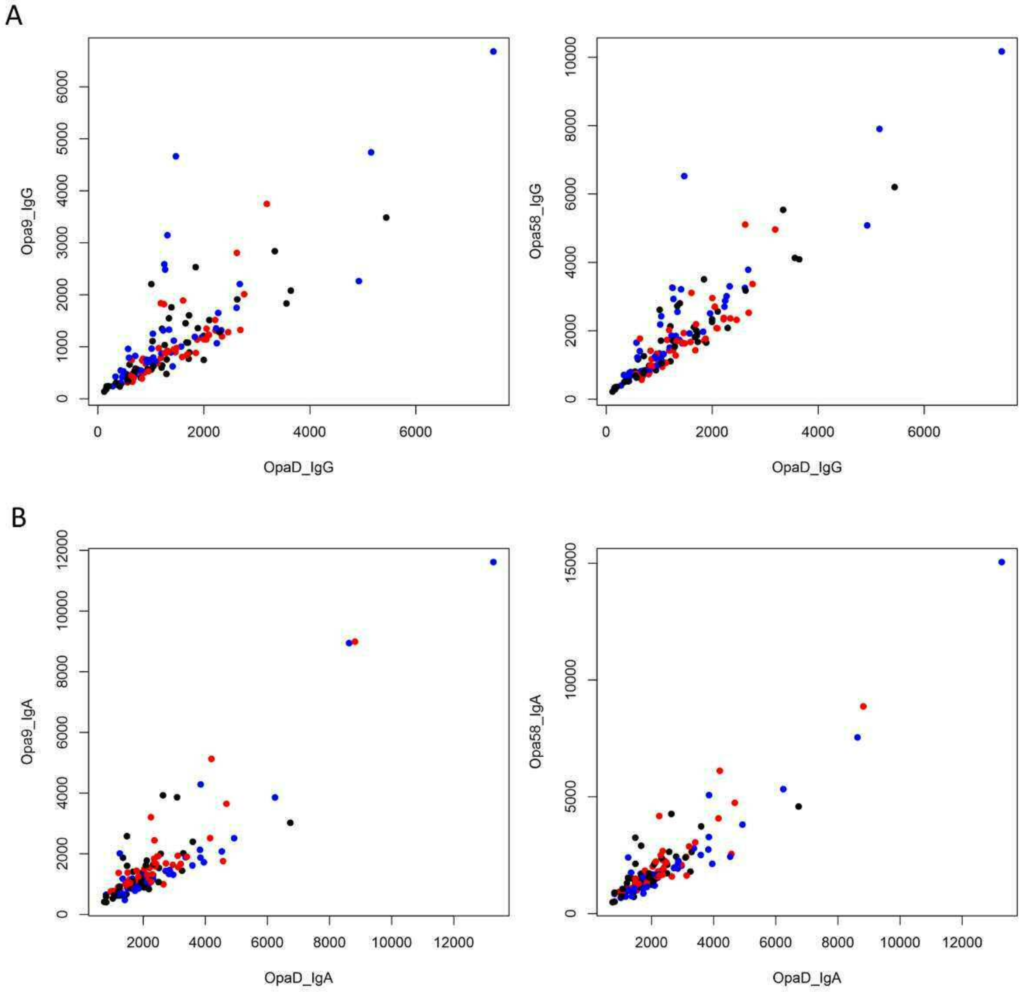

Figure S5 PCA analysis applied to IgG and IgA immunoprofiles in the vaccinated cohort at the 10 weeks timepoint. For each antigen, the increase in IgG or IgA response was calculated by subtraction of the 0 week timepoint (t0) from the 10 week timepoint (t10). PCA analysis was then carried out using the matrix of antigen responses (t10-t0) for each vaccinated individual (n=47) Points are coloured according to HIV status (positive/negative) or Sex (Female/Male). Ellipses are plotted for each group at 95% significance level, as implemented in factoextra. The centre of each ellipse is also included, as a larger symbol.

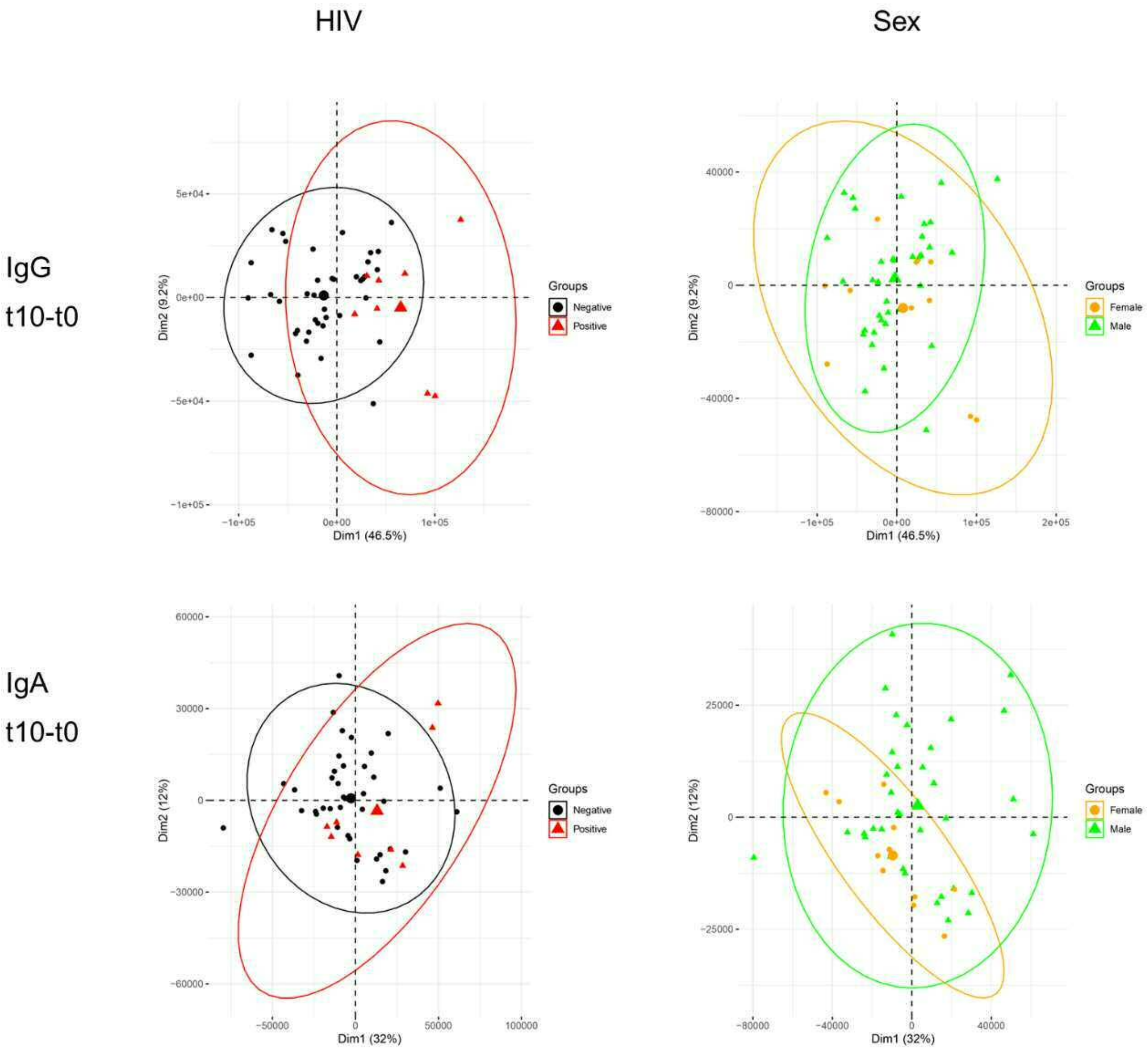

Figure S6 PCA analysis applied to IgG and IgA immunoprofiles in the vaccinated cohort at the 24 weeks timepoint. For each antigen, the increase in IgG or IgA response was calculated by subtraction of the 0 week timepoint (t0) from the 24 week timepoint (t24). PCA analysis was then carried out using the matrix of antigen responses (t24-t0) for each vaccinated individual (n=47). Points are coloured according to HIV status (positive/negative) or Sex (Female/Male).

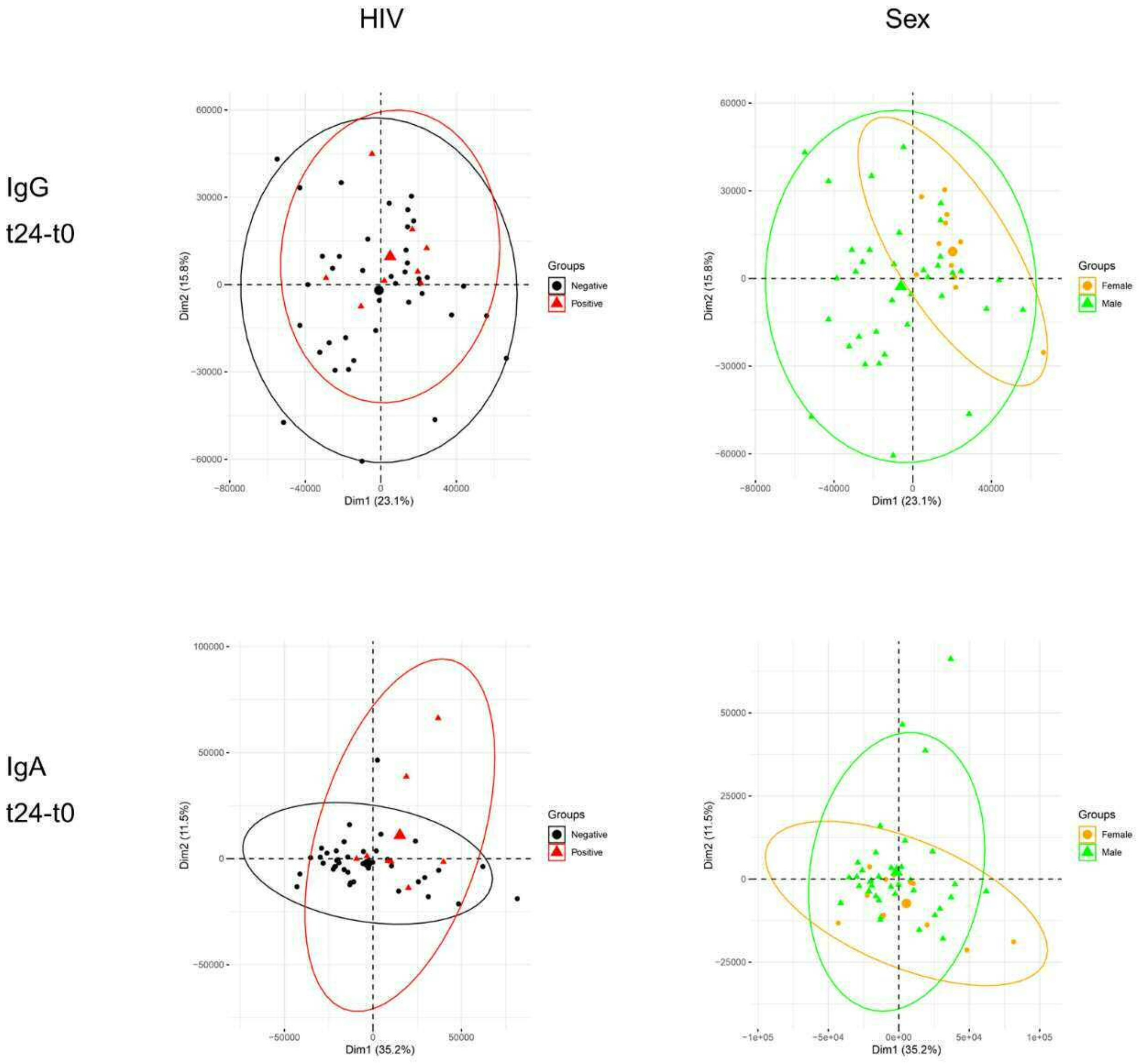

Figure S7 Violin plots of bactericidal activity (% recovery) at three serum dilutions. Box plots are superimposed on each violin plot, providing the median, two hinges which correspond to the first and third quartiles and ‘whiskers’ which extend to the ‘outlier’ points, which are plotted explicitly. n=47, for t0, t10 and t24. Low dilution: 1:480; Medium dilution: 1:960; High dilution: 1: 1,920.

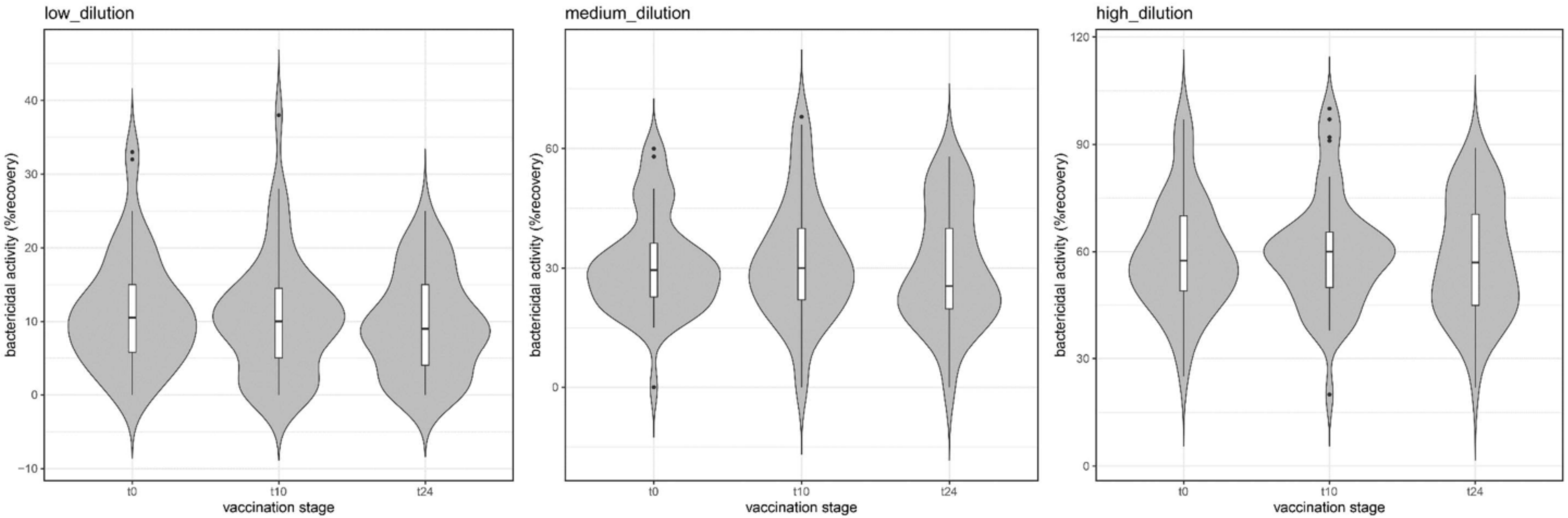

Figure S8 Violin plots of ELISpot activity. IFNγ-Spot-Forming Units per 10^6 PBMC are plotted for each of the pooled peptides detailed in Table S1. Box plots are superimposed on each violin plot, providing the median, two hinges which correspond to the first and third quartiles and 'whiskers' which extend to the 'outlier' points, which are plotted explicitly. n=47, for t0, t10 and t24.

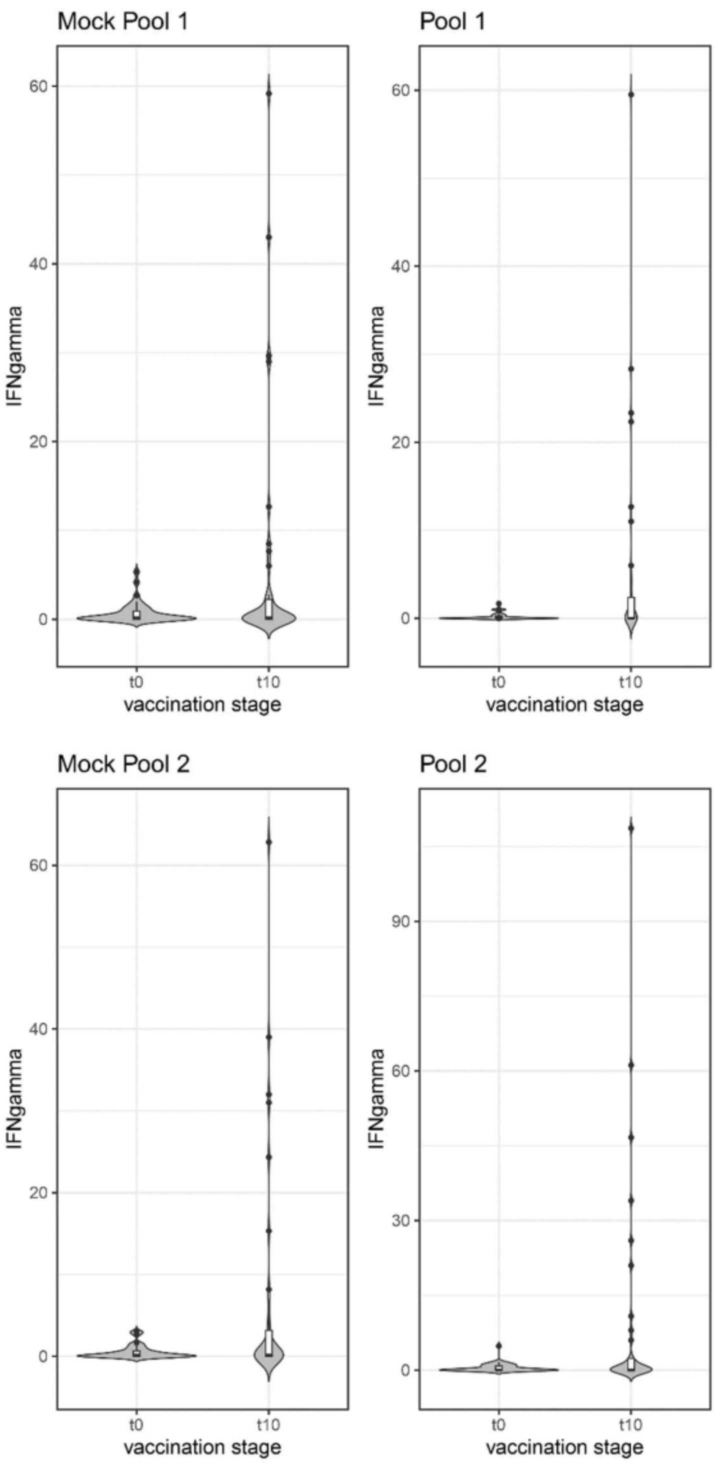

Table S1 Peptides used in ELISpot assays

Antigen	Name	Sequence	Name	Scrambled Sequence	Peptide Pool
NHBA	NHBA1	DFGRTNVGNSVVIDG	NHBA1sc	GGDTFSVVNVIDGNR	1
NHBA	NHBA2	AKGEMLVGTAVYNGE	NHBA2sc	NGLVKMYEAAEGGTV	1
PorB	PorB1	ADKIVSTASAVVLRHKF	PorB1sc	IKAAASVVFRLVKHDST	1
PorB	PorB2	KRTSALVSAGWLQRG	PorB2sc	SKRTAWLASGGQVLR	1
PilQ	PilQ	TDRRELLIFITPRII	PilQsc	RIEIPTLFIRDTIRL	2
NspA/Opa	NspA1	RGFYVQADAAHAKAS	NspA1sc	DFARYAAHAKQVSAG	2
BamA	BamA1	GPMKFSYAYPLKKKPE	BamA1sc	SKPKLGAKYPMPEFYK	2
NspA	NspA2	RINDLRFAVDYTRYK	NspA2sc	AVRDLTYRDNKIYRF	2

Clinical Trial Protocol

KWTRP
CLINICAL TRIAL PROTOCOL

Use of Bexsero immunisation to detect cross reactive antigens and anti-gonococcal antibodies in key populations in Kenya

1. GENERAL INFORMATION

**Protocol Number:**	**CSC 182**
**Trial Registration Number:**	ClinicalTrials.gov ID: NCT04297436
**Investigational Product(s):**	4CMenB vaccine (Bexsero®)
**Funder:**	**Wellcome Trust**,<br>215 Euston Road<br>London NW1 2BE, UK
**Tel:**	+44 2076 118888
**Email:**	s.towers@wellcome.ac.uk
**Sponsor:**	**University of Oxford**<br>University Offices, Wellington Square, Oxford OX1 2JD
**Tel:**	+44 1865 572245
**Email:**	ctrg@admin.ox.ac.uk
**Sponsor's authorized representative /investigator**	Prof. Eduard Sanders<br>PO Box 239, Kilifi 80108
**Tel:**	+254723593-762
**Email:**	ESanders@kemri-wellcome.org
**Study monitor:**	KWTRP- Clinical Trials Facility
**Email:**	MMunene@kemri-wellcome.org
**Statistician:**	Benedict Orindi<br>PO Box 239, Kilifi 80108
**Email:**	Borindi@kemri-wellcome.org
**Central Laboratory:**	KEMRI-Wellcome Trust Research lab's
**Tel:**	+254 709 983000
**Email:**	info@kemri-wellcome.org
**Drug/Product Manufacturer:**	4CMenB vaccine (Bexsero® -GSK)<br>980 Great West Road, Brentford<br>Middlesex, TW8 9GS
**Tel:**	Tel: +44 20 8047 5000
**Email:**	customercontactuk@gsk.com

Confidentiality Statement

The information contained herein is privileged or confidential and may not be disclosed unless such disclosure is required by applicable laws or regulations. In any event, persons to whom the information is disclosed must be informed that the information is privileged or confidential and may not be further disclosed by them. These restrictions on disclosure will apply equally to all future information supplied to you, which is indicated as privileged or confidential. This confidentiality statement also applies to data generated during the course of the study.

SPONSOR'S APPROVAL OF THE PROTOCOL

Use of Bexsero immunisation to detect cross reactive antigens and anti-gonococcal antibodies in key populations in Kenya

Protocol Number:

The following personnel have reviewed and approved this protocol **Bexsero trial in key populations**, version number 1.3, and date:

Signature:

Dr. Rebecca Bryant, Research Ethics Manager Date
OxTREC

INVESTIGATOR'S APPROVAL OF THE PROTOCOL

Use of Bexsero immunisation to detect cross reactive antigens and anti-gonococcal antibodies in key populations in Kenya

Protocol Number: CS 182

The undersigned acknowledge possession of and have read the Investigators' Brochure, Edition x, month year, and protocol **Bexsero trial in key populations** version 1.4 dated 6 November 2020. Having fully considered all the information available, the undersigned consider that it is ethically justifiable to give 4CMenB vaccine (Bexsero® to selected participants according to the agreed protocol.

I understand that all information concerning 4CMenB vaccine (Bexsero®) supplied to me by University of Oxford and/or its agents in connection with this study and not previously published is confidential information. This includes the Investigators' Brochure, Clinical Trial Protocol, Case Report Forms and any other preclinical

and clinical data provided by University of Oxford. I understand that no data are to be made public or published without prior knowledge and written approval by University of Oxford.

By my signature below, I hereby attest that I have read, understood and agreed to abide by all the conditions, instructions and restrictions contained in Protocol **Bexsero trial in key populations** version 1.4 dated 6 November 2020 and in accordance with the most recent Declaration of Helsinki and Good Clinical Practice and all applicable regulatory requirements.

I acknowledge that the Sponsor of the study University of Oxford has the right to discontinue the study at any time.

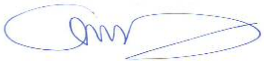

6 November 2020

Chief Investigator Signature **Date**

Prof. Christoph Tang, Professor of Pathology,

Chief investigator, Gonococcal Vaccine Initiative

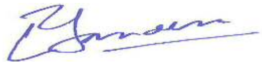

6 November 2020

Principal Investigator Signature **Date**

Prof. Eduard Sanders, principal investigator

Table of Contents

1. GENERAL INFORMATION ... 1
GLOSSARY OF TERMS AND ABBREVIATIONS: ... 6
2. LAY SUMMARY ... 7
3. LIST OF INVESTIGATORS ... 8
4. ABSTRACT ... 9
5. INTRODUCTION ... 9
5.1. Background Information ... 9
5.2. Name and description of the investigational product(s), ... 13
5.3. Justification ... 14
6. TRIAL OBJECTIVES AND PURPOSE ... 14
6.1. Null hypothesis ... 15
7. TRIAL DESIGN ... 15
7.1. Overall Study Design and Plan Description ... 15
7.1.1. Recruitment ... 16
7.1.2. Screening ... 17
7.1.3. Enrolment ... 17
7.1.4. Unscheduled (interim) Visits ... 18
7.1.5. End of Treatment Visitt ... 18
7.1.6. **Follow-up Visit(s)**. ... 19
7.1.7. Study Restrictions ... 19
7.2. Choice of Control Groups ... 20
8. SELECTION AND WITHDRAWAL OF STUDY PARTICIPANTS ... 20
8.1. Description of the population to be studied ... 21
8.2. Inclusion criteria ... 21
8.3. Exclusion criteria ... 21
8.4. Withdrawal criteria ... 21
8.5. Managing withdrawals ... 22
8.6. Replacing withdrawn participants ... 22
9. TREATMENT OF STUDY PARTICIPANTS ... 22
9.1. Treatments ... 22
Identity of Investigational Product (IP) ... 22
9.2. Storage ... 23
9.3. Dose Selection ... 23
9.4. Timing of Doses ... 23
9.5. Randomisation and Blinding ... 23
9.6 Packaging and Labelling. ... 23
9.7. Dispensing Procedures ... 23
9.8. Dose Administration ... 23
9.9. Accountability ... 23
9.10. Unblinding ... 23
9.11. Treatment Compliance ... 23
9.12. Overdosage. ... 23
9.13. Prior/Concomitant Therapy ... 23
9.13.1. Prohibited Medication ... 23
9.13.2. Rescue Medication ... 23
10. ASSESSMENT OF IMMUNOGENICITY ... 23
10.1 Identify *Ng* cross-reactive, human immune responses elicited by Bexsero®: ... 24
10.3 Determining Th1 proliferation ... 24
Antibody Responses ... 25
Sample storage: ... 26
10.7. Drug concentration measurements ... 26
10.8. Health economic measures ... 27
11. ASSESSMENT OF SAFETY ... 27

11.1. Adverse Events (AEs) 27
11.2. Definitions and monitoring of AEs 27
11.3. **Causality** 28
11.4. Procedures for Recording Adverse Events 29
Documenting AEs 29
Reporting Serious Adverse Events (SAEs) and/or Unexpected AEs 29
11.5. Emergency Procedures 31
11.3. Pregnancy 31
12. STATISTICS 31
12.1. Determination of sample size 31
12.2. Statistical and analytical plans 32
13. Access to Data 32
14. Data Recording and Record Keeping 33
15. Data entry at study site 33
15.1.1. Planned statistical methods 33
16. DIRECT ACCESS TO SOURCE DATA/DOCUMENTS 34
17. QUALITY CONTROL AND QUALITY ASSURANCE 34
17.1. **Choice of investigators** 35
17.2. Monitors and monitoring plan 35
18. INTELLECTUAL PROPERTY 36
19. TIME FRAME/DURATION OF THE TRIAL 37
20. ETHICS 37
20.1. Human Subjects 37
20.2. Community Considerations 39
20.3. Informed Consent 40
20.4. Compensation 40
20.5. Patient Data Protection/Confidentiality 40
20.6. Data Sharing 40
20.7. Safety 41
20.8. Material Transfer Agreement (if applicable) 41
21. ARCHIVING AND RECORD RETENTION 42
22. FINANCING AND INSURANCE 42
22.1. Budget 42
22.2. Insurance 43
23. TRIAL MANAGEMENT 44
24. REPORTING, DISSEMINATION AND NOTIFICATION OF RESULTS 44
25. APPENDICES 45
25.1. Roles of Investigators 45
REFERENCES 45

GLOSSARY OF TERMS AND ABBREVIATIONS:

AMR	antimicrobial resistance
ART	anti-retroviral therapy
CRF	Case record form
CDC	Centers for Disease Control and Prevention
C4BP	C4 binding protein
CT	Chlamydia trachomatis
fH	factor H
fHbp	Factor H binding protein
FSW	Female sex workers
Gc	Gonococcal
HBV	Hepatitis B virus
iOMP	integral outer membrane protein
LGBT	Lesbian Gay Bisexual Transgender
MenB	Meningococcal B
MSW	Male sex workers
MSM	men who have sex with men
NadA	Neisseria adhesin A
Ng	Neisseria gonorrhoeae
NHBA	Neisserial Heparin binding antigen
Nm	Neisseria meningitidis
OMV	outer membrane vesicles
POC	point-of-care
PorA P1.4	Porin A
PrEP	Pre-exposure Prophylaxis
RmpM	reduction-modifiable protein M
STI	sexually transmitted infections
Th1	T helper cell 1
WHO	World Health Organization

2. LAY SUMMARY

Formal Title Use of Bexsero immunization to detect cross-reactive antigens and anti-gonococcal antibodies in key populations in Kenya

Lay Title Gonococcal vaccine study in key populations in Kenya

What is the problem/background? Gonorrhoea is a sexually transmitted infection that can infect both men and women. It can cause infections in the genitals, rectum, and throat. It is a very common infection, especially among young people aged 18-25 years. Meningococcal disease and gonorrhoea are caused by bacteria that are closely related but cause different diseases that are spread in different ways. New evidence suggests that the Meningococcal B vaccine (Bexsero®) licensed outside of Kenya against meningococcal B disease may also be effective against gonorrhoea due to genetic similarities between the two organisms causing the two diseases. The aim of this study is to generate data to develop a gonorrhoea vaccine, using an existing vaccine against meningococcal disease.

What questions are we trying to answer?

To assess if immunisation of individuals at risk for gonococcal infection with 4CMenB (Bexsero®) will enhance an immune response against *Neisseria gonorrhoeae.*

Where is the study taking place, how many people does it involve and how are they selected?

Approximately 50, young adults (aged 18 - 25 years old) who are enrolled in the KEMRI cohort studies (known as Open B, Three-site, N, or Sue; including HIV-uninfected and infected individuals) in the KEMRI clinic in Mtwapa or Malindi will be able to participate in this study. The study will take place at the KEMRI clinic in the Malindi sub-county hospital.

What does the study involve for those who are in it?

Participants will make five study visits, including a screening visit, an enrolment visit and 3 follow up visits. We will offer vaccination with the Meningococcal B vaccine (Bexsero®) vaccine at the enrolment visit and approximately 2 months later. We will use socio-demographic and risk behavior data collected under the KEMRI cohort studies. For participants living with HIV, we will collect a screening sample (1 tea spoon) and confirm that their viral load<200 copies/ml. We will collect a 24 ml blood sample (2 ½ table spoons) at enrolment, and a 70 ml blood sample (7 table spoons) following the second vaccination. At study completion (month 6), we will collect a 4ml (1 tea spoon) blood sample. We will collect a throat swab, a urine sample (for men), vaginal swab, and an anal swab at the enrolment, month 3 and month 6 visit to test for Chlamydiae and Gonorrhoea infection.

What are the benefits and risks/costs of the study for those involved? In this study, the Meningococcal B vaccine (Bexsero®) is safe and is well tolerated, with over 17 million doses already given worldwide. There is the possibility of common side effects that are mild and may include a mild fever, soreness at the injection site, headache and generally feeling unwell for 1-2 days, similar to some other vaccines. As with all vaccines there is the possibility of rare and more serious reactions such as an allergic/ anaphylactic reaction to the vaccine.

For participants there are no direct benefits, as they will receive the same research services as in the KEMRI cohorts that they participate in already. Meningococcal B vaccine (Bexsero®) prevents only against the meningococcal B strain which is common in Europe, Australia, and the Unites States.

How will the study benefit society? Researchers hope to gain new insights on how the Meningococcal B vaccine (Bexsero®) can be improved upon to make a vaccine against gonorrhoeae. There is currently no vaccine available against *N. gonorrhoeae*.

When does the study start and finish?
This study is anticipated to start in the fourth quarter of 2020 (or early 2021), following ethical and regulatory approvals. Participants will be involved in the study for 6 months. The sample and data analysis will continue for 1-2 years and a report will be disseminated in 2022.

3. LIST OF INVESTIGATORS

Prof. Christoph Tang	Chief investigator	University of Oxford
Prof. Eduard Sanders	Principal investigator	KEMRI-WTRP
Prof. Philip Bejon	co-investigator	KEMRI-WTRP
Dr. Eunice Nduati	co-investigator	KEMRI-WTRP
Dr. Clara Agutu	co-investigator	KEMRI-WTRP
Dr. Susan Graham	co-investigator	KEMRI-WTRP/ UW

4. ABSTRACT

Meningococcal disease and gonorrhoea are caused by bacteria that are closely related but cause different diseases that are spread in different ways. New evidence suggests that the Meningococcal B vaccine (Bexsero®) licensed outside of Kenya against meningococcal B disease may also be effective against gonorrhoea due to genetic similarities between the two organisms causing the two diseases. We will conduct a clinical trial of the Meningococcal B vaccine (Bexsero®) in approximately 50 male and female participants aged 18-25 years who are in follow up in KEMRI cohorts (including HIV-uninfected and infected individuals) at the KEMRI clinic in Mtwapa or Malindi. This is not an efficacy trial. Instead, we will assess if immunisation of individuals at risk for gonococcal infection with 4CMenB (Bexsero®) elicits humoral and T cell cross-reactive responses against Neisseria gonorrhoeae (Ng). Participants will make five study visits, including a screening visit, an enrolment visit and 3 follow up visits. We will offer vaccination with the Meningococcal B vaccine (Bexsero®) vaccine at the enrolment visit and approximately 2 months later. We will collect a 20ml blood sample at enrolment, and a 70ml blood sample following the second vaccination. At study completion (month 6), we will collect a 4ml blood sample. We will collect a throat swab, a urine sample (for men), vaginal swab, and an anal swab at the enrolment, month 3 and month 6 visit to test for Chlamydiae and Gonorrhoea infection. Total study participation for participants is 6 months. Upon study completion, participants will continue to receive research care at the KEMRI clinic in the Mtwapa-area and Malindi.

5. INTRODUCTION

5.1. Background Information

Infection with *Neisseria gonorrhoeae* (Ng) remains a major public health problem worldwide [1]. The bacterium is a leading cause of sexually transmitted infections (STI), and responsible for mucosal infection, cases of pelvic inflammatory disease [2], and chronic, asymptomatic infection [3]. Ng has a significant adverse impact on reproductive health (through ectopic pregnancy, infertility, prostatitis), while infection during birth can result in neonatal blindness [3]. Furthermore, Ng is an important risk factor for HIV acquisition, associated with a 4.6 fold increased risk [4]. There are over 300,000 cases of

Gonococcal (Gc) infection in the USA per annum, and the number of cases has risen dramatically in England (14,985 in 2008 to 42,4420 in 2017, https://www.gov.uk/government/statistics/sexually-transmitted-infections-stis-annual-data-tables), while the highest rates are in countries where diagnostic facilities and treatment are limited [5].

The relentless rise of antimicrobial resistance (AMR) in Ng, with resistance developing soon after antibiotics are introduced [6], poses challenges for effective therapy [7]. Furthermore, the emergence and spread of Ng strains which are resistant to azithromycin and ceftriaxone jeopardise control based on contact tracing and treatment of asymptomatic carriers [3]. Consequently, there is an urgent need to develop novel approaches to tackle Gc infection.

Historically, vaccines have proven remarkably effective in protecting individuals from infectious diseases, and can interrupt AMR spread, evident from the effect of conjugate capsular vaccines on *Streptococcus pneumoniae* [8].

There has been remarkably little progress in Ng vaccine development, even with recent advances in vaccine development against the closely related *Neisseria meningitidis* (Nm). The stumbling blocks for vaccines against Ng have been well documented: i) there is little evidence of immunity following natural infection[9], as the bacterium suppresses immune responses[10, 11]; ii) there are no known correlates of protection; iii) clinical trials with Gc killed whole-cell preparations offered no protection [12], while vaccines based on the pilin of Type IV pili, a major Gc adhesin were undermined by the emergence of strains expressing variant pilins in immunised volunteers [13], highlighting iv) difficulties in vaccine development with this variable pathogen.

However, technical advances and understanding of Ng immunity and pathogenesis provide strategies towards Gc vaccine development with prospects for success. Ng and Nm recruit the negative regulator of complement, factor H (fH)[14, 15]. Ng binds fH via PorB, the most abundant integral outer membrane protein (iOMP). There are two main variants of PorB, PI.A and PI.B, with the 5th extracellular loop of

PI.A engaging fH[14]; this is enhanced by sialylation of bacterial lipopolysaccharide[16]. PI.A and PI.B PorB loops also bind to C4 binding protein (C4BP), further subverting complement[17]. Furthermore, PorB is delivered to phagocytes in outer membrane vesicles (OMVs), causing apoptosis[18], potentially preventing antigen presentation. Additionally, antibodies against PIII (homologue of Nm RmpM) subvert responses against other surface antigens[19, 20].

OMV vaccines have been successfully deployed against clonal outbreaks of Nm. Nm epidemics have been controlled by OMV vaccines in Cuba, Norway, and most recently New Zeeland [21].

We will employ OMV-based approaches against Ng. Immunisation with Nm OMVs was associated with protection against Ng. An Nm outbreak in New Zealand led to vaccination with MeNZB, an OMV vaccine from the causative isolate NZ98/254. A recent case-controlled study estimated MeNZB effectiveness against Ng as 31% (C.I. 21-39) [22]. The basis of this cross-protection is assumed to be similarity of Ng and Nm surface proteins. OMVs from NZ98/254 are a key constituent of Bexsero®, licensed Nm vaccine given in the UK[23]. Therefore, we exploit Bexsero® as an immunological probe to identify Ng antigens by cross-reactive responses in individuals with high Gc exposure as recurrent exposure to Ng may elicit some immunologic memory [24].

A recent study assessed cross reactivity to Ng of serum raised to the 4CMenB vaccine (Bexsero®), which contains the MeNZB OMV component plus three recombinant antigens (NadA, fHBP-GNA2091, and NHBA-GNA1030).[25] It was shown that a high level of sequence identity exists between MeNZB OMV and Bexsero OMV antigens, and gonococcal proteins. NHBA is the only 4CMenB vaccine (Bexsero®) recombinant antigen that is conserved and surfaced exposed in Ng. 4CMenB vaccine (Bexsero®) induces antibodies in humans that recognise gonococcal proteins.[25] The goal of this study is to define the immune responses to Bexsero® in individuals (HIV uninfected and infected) who are at risk and are likely to have been exposed previously to *N. gonorrhoeae*. This is with the overall aim of generating a vaccine against *N. gonorrhoeae* by identifying cross-reactive

antigens elicited by the meningococcal vaccine Bexsero® that might confer protection against gonococcal infection. In this way, we will be exploiting a vaccine that has already been licensed in the UK and the US for use in our target population. Therefore this is not an efficacy study, and we will not require a control group with non-immunised subjects.

Description of the population to be studied

Trial participants will be recruited from a KEMRI-cohort of mostly male and female sex workers (MSW; FSW) in Mtwapa and Malindi, which represents an at-risk, core population for STI transmission. Our incidence estimates for Ng (based on Xpert testing) in HIV-negative 18-25 year-old MSM participants is 21.4 per 100 person-years (95% CI: 13.1-35.0), and 43.5 per 100 person-years (95% CI: 23.4-80.8) in HIV-infected individuals (unpublished). At present, the KEMRI cohort in Mtwapa follows 125 MSM aged 18-25 years (100 HIV-1 negative, and 25 HIV-1 positive), and 9 FSW (5 HIV-1 negative-, and 4 HIV-1 positive). There is no Ng incidence estimates available for women. Therefore, this cohort is subject to high Ng exposure, so study participants are likelier to develop responses against Ng than the general population [24].

Summary of the known and potential risks and benefits, if any, to human participants

Potential Benefit to Participants: Vaccine recipients will have no direct benefit of receiving 4CMenB vaccine (Bexsero®) as Meningococcal B (MenB) disease is uncommon in Kenya. Participants will benefit from point-of-care (POC)X-pert sexually transmitted infections (STI)screening and treatment of infections as most STI are asymptomatic. All participants in the KEMRI cohort studies benefit from regular risk reduction counselling, HIV testing (when HIV negative), hepatitis B virus (HBV) vaccination, pre-exposure prophylaxis (PrEP) (if participants indicate that they wish to be on PrEP), and antiretroviral therapy (ART).

Potential Risk to Participants:

1. Collection of oropharyngeal and anal swabs for STI screening. Collection of swabs can be uncomfortable, but should cause minimal distress to participants. **2.** Vaccination.
The most common reported side effect in adolescents and young adults following vaccination with 4CMenB (Bexsero®) is pain at the injection site, headache, and generally feeling unwell. Other possible side effects include fever, feelings of tiredness and nausea [26].

As with all vaccines, there is a small chance of an allergic reaction to the vaccine including a severe allergic reaction, or anaphylaxis (risk less than 1 in a million doses for existing vaccine) [27].

5.2. Name and description of the investigational product(s),

4CMenB vaccine (Bexsero®) was licensed in 2013 in Europe and North America and in various other jurisdictions. 4CMenB vaccine (Bexsero®) is an FDA-approved vaccine to prevent invasive disease caused by Neisseria meningitidis serogroup B. It is approved for use in individuals 10 through 25 years of age. During clinical development, the vaccine was evaluated in adolescents, and it was demonstrated that two doses of 4CMenB vaccine (Bexsero®) induced robust immune responses against the vaccine antigens [26, 28]. The vaccine was well tolerated, and no safety concerns were identified (reviewed in [29]).

<u>Route of administration, dosage, dosage regimen, and vaccination period</u>

4CMenB vaccine (Bexsero®; 0.5mL). The vaccine is given by deep intramuscular injection, in the deltoid muscle region of the upper arm, twice and approximately two months apart. Each dose of vaccine contains recombinant Neisseria meningitidis group B NHBA fusion protein (50 micrograms); recombinant Neisseria meningitidis group B NadA protein (50 micrograms); recombinant Neisseria meningitidis group B fHbp fusion protein (50 micrograms) and Outer membrane vesicles (OMV) from Neisseria meningitidis group B strain NZ98/254 (25 micrograms measured as amount of total protein containing the PorA P1.4) adsorbed on aluminium hydroxide (0.5 mg $Al3+$).

5.3. Justification

Neisseria gonorrhoeae (Ng) is a leading cause of sexually transmitted infections (STI) worldwide, responsible for >70 million cases annually [30]. The emergence of antimicrobial resistance (AMR) has made combatting Ng a priority for the WHO and CDC. Difficulties treating Ng adversely impacts female reproductive and foeto-maternal health, while gonococcal (Gc) infection is an important cofactor for HIV transmission [31]. Furthermore, control of Gc transmission is based on contact tracing and treatment, and threatened by AMR [32].

While vaccines are a valuable approach to combat AMR, Gc vaccine research has been hampered by limited/lack of protective immunity following infection, no correlates of protection, variation of Ng, and past vaccine failures.

However, recent advances have provided fresh impetus for vaccine development. Our collaborators will validate effective candidate antigens for inclusion in a Gc vaccine through pre-clinical evaluation, work that falls outside of this protocol. Our study will assess immune responses in highly exposed individuals before and after vaccination with Meningococcal B vaccine (Bexsero®). Previously, Plummer et al showed that female sex workers with repeated NG infection in Nairobi had specific but incomplete protection against subsequent infection with the homologous serovar.[24] For this reason, we will enroll highly exposed individuals from Kenya. Specifically, we aim to identify Ng cross-reactive, human immune responses elicited by 4CMenB vaccine (Bexsero®): We will perform a clinical trial of Bexsero®, a licensed vaccine outside of Kenya containing *Neisseria meningitidis* (Nm) OMVs, in a well characterised, at-risk cohort of sex workers in Kenya. We do not intend to evaluate the protective efficacy of 4CMenB vaccine (Bexsero®). Instead we will exploit 4CMenB vaccine (Bexsero®) as an immunologic probe to identify antigens that elicit humoral and T cell cross-reactive responses against Ng, which will inform vaccine design.

6. TRIAL OBJECTIVES AND PURPOSE

6.1. Null hypothesis. This is an explorative study using 4CMenB vaccine (Bexsero®). Therefore, a null hypothesis will not be tested.

6.2 Primary objective –To assess if immunisation of individuals at risk for gonococcal infection with 4CMenB (Bexsero) elicits humoral and T cell cross-reactive responses against Ng.

Explorative objective – To assess if immunisation of individuals at risk for gonococcal infection with 4CMenB (Bexsero) differs between HIV negative and positive participants.

We will exploit Bexsero as an immunological probe to identify antigens that elicit humoral cross-reactive responses against Ng. We will examine IgG reactivity against these Ng proteins in microarrays with sera from Bexsero vaccinated individuals with high Ng exposure.

Comparison of the pre/post immunization sera will provide a comprehensive overview of Bexsero antigens which induce Ng-cross reactive IgG responses and could therefore be responsible for Nm OMV-mediated protection against Ng. This approach is likely to be more informative than comparing results between control and immunized subjects, as individuals' exposure and responses to Ng are likely to differ. We will also explore if immune responses differ between HIV-negative and positive participants. Screening will be performed on plasma taken at enrolment, approximately 2 weeks post first immunization, approximately 2 weeks post second immunization and at 6 months.

7. TRIAL DESIGN

7.1. Overall Study Design and Plan Description

This study will be an open-label single-arm clinical trial with a 4CMenB vaccine (Bexsero®), licensed in over 40 countries. The study will take place at the KEMRI-clinic in Malindi which has provided HIV prevention and care services to key populations since 2010. Prospective participants will be invited from existing prevention and care cohort studies. Therefore, all participants will be in regular (3-monthly) follow up, have received hepatitis B vaccination, receive HIV testing (if HIV negative), and adherence counselling for PrEP or ART, as appropriate. The KEMRI clinic is supported by a

community engagement programme, and new studies will be discussed with a key population community advisory board, and other stake holders.

Trial participants will be aged 18-25 years, be available for follow up for six months, and not participate in any other vaccine study. A course of Bexsero® vaccinations requires a minimum interval of one month. Participants will be immunised twice at an interval of two months to allow for maturation of immune responses. Participants will be screened for Chlamydia trachomatis (Ct) and Ng in urine/vaginal, rectal and oropharyngeal secretions at enrolment, month 3, and month 6 (study completion), and receive treatment when a CT/Ng infection is detected. We will collect approximately 6 ml at study screening (for participants living with HIV only), 20 ml blood at enrolment, 70 ml of blood two weeks after the second vaccination, and 4 ml at study completion.

Table 1: Study Schedule

	**Visit 1 Screening**	**Visit 2 Enrolment**	**Visit 3 Follow-up**	**Visit 4 Follow-up**	**Visit 5 Follow-up**
**Study week**	**-2**	**0**	**8**	**10**	**24**
**Assessment / Procedure**					
Informed Consent	x				
Demography	x				
Medical history	x				
Symptom directed physical examination		x		x	x
Urine (pregnancy test)		x	x		
Vaccination		x	x		
Blood /swab collection for lab assays and storage	x	x		x	x

7.1.1.Recruitment

Healthy adult male and female volunteers, aged 18-25 years, participating in ongoing KEMRI cohorts (SSC 894, SSC1224, SERU 3520, SERU 3788) at the Mtwapa or Malindi clinics will be eligible for

study screening. The KEMRI cohorts SSC No. 894 and SERU 3788 follows HIV negative at-risk participants who are in three-monthly follow up, and receive risk reduction counselling, PrEP, and treatment for symptomatic STI's. KEMRI cohort SERU No. 3520 and SSC 1224 follows HIV-positive participants in monthly and three-monthly follow up, respectively, who receive risk reduction counselling, ART, and treatment for symptomatic STI's. HIV-positive participants who are adherent to ART and virally suppressed (i.e. viral load < 200 copies per ml) are considered healthy.

We will aim to enroll both HIV-negative and positive participants, mostly male and female sex workers, and target participants for whom Ng was detected by Xpert CT/NG testing during their previous KEMRI visits. At present, over 100 HIV-negative participants (100 MSM, 5 FSW), and 29 HIV-positive participants (25 MSM and 4 FSW) aged 18-25 years are eligible for screening.

Prior to study screening, participants will be invited to an information session at the KEMRI clinic and their interest in study participation assessed.

7.1.2.Screening

Screening assessments will be performed up to 14 days before enrolment. Screening procedures will only be performed after the volunteer's written consent has been obtained. Before providing informed consent, prospective volunteers will receive a study screening number and be screened for study eligibility. Demographic, risk behaviour and medical history information will be obtained from existing protocol databases. For participants living with HIV, a 6 ml sample will be collected to confirm that the viral load<200 copies per ml to be eligible for screening.

7.1.3.Enrolment

All eligible participants will receive a vaccination at their enrolment visit.

Prior to the first vaccination, site personnel will:

- Review any questions the volunteer may have about the study
- Review the Informed Consent Document with the volunteer

- Review screening safety laboratory data (i.e. pregnancy screen)
- Review interim medical history
- Assess baseline vital signs (i.e. axillary temperature)
- Perform a symptom directed physical examination
- Collect specimens for all tests as indicated in the Schedule of Procedures Appendix A, including CT/NG testing of oropharyngeal and anal swabs, and urine.
- Administer the 4CMenB vaccine (Bexsero®) according to instructions in the Study Operations Manual.
- Observe volunteer closely for at least 30 minutes after vaccine/investigational medicinal product administration for any acute (reactogenicity/solicited adverse reactogenicity /solicited adverse reactions).
- Assess any other adverse events

7.1.4. Unscheduled (interim) Visits

Visits / contacts other than those described in the Schedule of Procedures (Appendix A) will be classified as unscheduled or interim visits and recorded on applicable source documents. They may occur:

- For administrative reasons e.g. the volunteer may have questions for the study staff or may need to re-schedule a follow up visit.
- To review a laboratory investigation from a previous visit
- To review the outcome of an adverse event.
- To conduct a study visit where a volunteer has missed the scheduled study visit window.
- For any other reason requested by the volunteer or Principal Investigator.

7.1.5. End of Treatment Visit. Participants will have a second vaccination scheduled approximately two months after enrolment at which the first vaccination has been given **(s**chedule of Procedures -Appendix A)

Prior to the second vaccination, site personnel will:

- Review any questions the volunteer may have about the study
- Review the Informed Consent Document with the volunteer
- Review screening safety laboratory data and perform a pregnancy test
- Review interim medical history
- Assess baseline vital signs (i.e. axillary temperature)
- Perform a symptom directed physical examination
- Collect specimens for all tests as indicated in the Schedule of Procedures Appendix A
- Administer the 4CMenB vaccine (Bexsero®) according to instructions in the Study Operations Manual.
- Observe volunteer closely for at least 30 minutes after vaccine/investigational medicinal product administration for any acute (reactogenicity/solicited adverse reactogenicity /solicited adverse reactions).
- Assess any other adverse events.

7.1.6. **Follow-up Visit(s).** One follow up visit is scheduled approximately two weeks following receipt of the final 4CMenB vaccine (Bexsero®) according to the schedule of procedures, for blood collection and immunogenicity assessments, and a final follow up visit is scheduled 24 weeks post enrolment for CT/NG testing of oropharyngeal and anal swabs, and urine, and immunogenicity assessments. Upon detection of any CT/NG infection, participants will be contacted to receive treatment. Upon detection of any Xpert NG infection, swabs will be collected from the infected site for Ng culture and drug sensitivity testing.

7.1.7. Study Restrictions

Each participant has the right to withdraw from the trial at any time.

If a participant withdraws consent for the whole study, no further study procedures should take place. In some circumstances, participants will withdraw consent for specific procedures, or fail to attend a visit, but may still be willing to participate in subsequent visit/ procedure.

In this situation the following guidelines apply:

- If any of the procedures at the enrolment visit are refused then they will be considered as withdrawn from the study, and no further procedures will take place.
- If a participant refuses or doesn't attend for the 2nd immunisation, they can remain in the study for the final CT/NG swab and urine and immunogenicity blood collection, and should be encouraged to do so.

In addition, the Investigator may discontinue a participant from the trial at any time if the Investigator considers it necessary for any reason including:

- An adverse event which requires discontinuation of the trial medication or results in inability to continue to comply with trial procedures
- Withdrawal of Consent (as above)
- Loss to follow up, but we will still include any data already gathered from the participant in the final analysis, unless consent is withdrawn.
- Exclusion from the trial will not result in exclusion of the data already taken for that participant.
- The reason for withdrawal will be recorded in the case record form (CRF).
- If the participant is withdrawn due to an adverse event, the Investigator will arrange for follow-up visits or telephone calls until the adverse event has resolved or stabilised.
- Withdrawn participants will not be replaced.
- **Patient Obligations.** Participants are expected to complete all 5 study visits, and report any adverse event to the investigators.

7.2. Choice of Control Groups

n/a.

8. SELECTION AND WITHDRAWAL OF STUDY PARTICIPANTS

8.1. Description of the population to be studied

We will enroll up to 50 participants, including approximately 35 HIV-uninfected and 15 HIV-1 infected male and female sex workers from ongoing KEMRI observational studies.

8.2. Inclusion criteria

- Healthy male and female as assessed by a medical history, physical exam, and laboratory tests. HIV-positive participants who are adherent to ART and virally suppressed (viral load <200 copies per ml) are considered healthy.
- At least 18 years of age on the day of screening and will not reach 26th birthday on the day of the second vaccination (approximately 6 weeks after enrolment)
- Willing and able to give informed consent for participation in the trial before any study-related procedures are performed.
- Willing to donate blood samples for immunogenicity assessments.

8.3. Exclusion criteria

- Any clinically significant acute or chronic medical condition that is considered progressive that, in the opinion of the Principal Investigator or designee, makes the volunteer unsuitable for participation in the trial
- Pregnancy
- Participation in another clinical trial (i.e. investigational HIV vaccine candidate), within the previous 3 months or expected participation during the study
- Bleeding disorder diagnosed by a physician (e.g., factor deficiency, coagulopathy or platelet disorder that requires special precautions).
- History of severe local or systemic reactogenicity to vaccines (e.g., anaphylaxis, respiratory difficulty, angioedema).

8.4. Withdrawal criteria

The Final Visit or Early Termination Visit procedures will be performed according to the Schedule of Procedures (Appendix A). Participant retention strategies and procedures are aimed

to achieve a study retention of >90%. Participants may voluntarily withdraw from the study at any time and for any reason. Participants may be withdrawn from the study permanently if:

- The principal investigator(s) or designee has reason to believe that a participant is not complying with the study or study procedures.
- The sponsor or local regulatory authorities decide to stop or cancel the study.
- Participant is lost to follow up.

8.5. Managing withdrawals

- If a participant withdraws from the study, the date and reasons for study withdrawal (if available) will be recorded in the volunteer's source documents and study records and stored samples will be retained unless consent is withdrawn.

8.6. Replacing withdrawn participants

This is not an efficacy trial, but an observational study of immune responses to a licensed vaccine. We will not replace any participant should any participant withdraw, as our sample will oversample up to 10% of participants.

9. TREATMENT OF STUDY PARTICIPANTS

9.1. Treatments

4CMenB vaccine (Bexsero® -GSK) 0.5mL intra-muscularly; two doses administered 2 months apart. Each dose of vaccine contains recombinant *Neisseria meningitidis* group B NHBA fusion protein (50 micrograms); recombinant *Neisseria meningitidis* group B NadA protein (50 micrograms); recombinant *Neisseria meningitidis* group B FHbp fusion protein (50 micrograms) and Outer membrane vesicles (OMV) from *Neisseria meningitidis* group B strain NZ98/254 (25 micrograms measured as amount of total protein containing the PorA P1.4) adsorbed on aluminium hydroxide (0.5 mg Al^{3+}).

The vaccine will be administered into the deltoid muscle of the non-dominant arm.

Identity of Investigational Product (IP)

4CMenB vaccine (Bexsero® -GSK) - Bexsero suspension for injection in pre-filled syringe. The manufacturer will be requested to label the vaccine as an investigational product for clinical trial use only.

9.2. Storage

Supply of 4CMenB will be through GSK, UK. The vaccine will be stored in 2–8 degree Celsius fridges at KEMRI clinic study pharmacy. The fridge will be temperature monitored.

9.3. Dose Selection

There are no planned dose modifications.

9.4. Timing of Doses

n/a.

9.5. Randomisation and Blinding. *n/a.*

9.6 Packaging and Labelling. . Bexsero® (GSK vaccine) will be packaged and labelled by the manufacturer. We will ensure that the vaccine will be labelled as an investigational product for clinical trial use only.

9.7. Dispensing Procedures. Dispensing procedures will be described in the study operational manual (SOM).

9.8. Dose Administration. Vaccine will be provided in a pre-filled syringe**.**

9.9. Accountability

4CMenB vaccine (Bexsero® -GSK) will be ordered through GSK. Investigational medical product (IMP) will be administered according to the protocol. No additional labelling of IMPs is required.

9.10. Unblinding. *n/a.*

9.11. Treatment Compliance

Participants are expected to complete all 5 study visits, including a screening visit, 2 vaccination visits, a 2-week post vaccination visit, and an end of study visit.

9.12. Overdosage *n/a.*

9.13. Prior/Concomitant Therapy There are no concomitant medications that would result in exclusion from this trial, therefore we will not be recording them.

9.13.1. Prohibited Medication. *n/a.*

9.13.2. Rescue Medication. *n/a.*

10. ASSESSMENT OF IMMUNOGENICITY

10.1 Identify *Ng* cross-reactive, human immune responses elicited by Bexsero®:

We will exploit Bexsero as an immunological probe to identify antigens that elicit humoral cross-reactive responses against Ng. Our collaborators have analysed the OMV proteome of Bexsero and have constructed protein microarrays containing 91 *Nm* proteins. We will examine IgG reactivity against these Nm proteins in microarrays with sera from Bexsero vaccinated individuals with high Ng exposure at a collaborative laboratory at the University of Manchester.

Comparison of the pre/post immunization sera will provide a comprehensive overview of Bexsero antigens which induce Ng-cross reactive IgG responses and could therefore be responsible for Nm OMV-mediated protection against Ng. Screening will be performed on plasma taken at enrolment, approximately 2 weeks post second immunization and at 6 months time point. The screening will take place at three sites, KEMRI –CGMRC, the University of Oxford, and a collaborators lab at the Uniformed Services University in the USA. Antigens and/or microarrays will be sent to KEMRI-CGMRC where printing on slides and running of the microarray will be performed. Microarrays will also be done by Jeremy Derrick at University of Manchester. Samples with cross-reactive antibodies against the OMV antigens, which may offer protection against Ng will be selected to inform antigen choice and vaccine design.

10.2 Determining systemic antibody levels and subclass analysis

Serum antigen- and OMV-specific IgG1, IgG2a, Ig3A and IgA titres will be determined by ELISA approximately two weeks after final immunizations. Responses will be compared among pre-post test groups by repeated measures ANOVA.[33]

10.3 Determining Th1 proliferation

Currently all candidate vaccines that show efficacy in the mouse models against Ng (2C7 peptide mimetic, Gc OMVs administered with IL-12) induce Th1 responses compared to over 10 other Gc antigens that were tested with Th2-inducing adjuvants and were not protective. T cell responses will be measured in two ways:

Indirect responses: by determining the IgG1/IgG2 ratios after immunization. Ig subclass levels (including IgG1/IgG2a ratios) will be indicative of Th1 responses.

Direct responses: PBMCs will be isolated at enrolment, , and 2 weeks post second immunization and used for the detection of antigen-specific IFNγ secreting T cells. A panel of overlapping peptides that cover the entire length of the immunogen will be used. To maximize cell use, IL-2 and IFNγ will be simultaneously detected using a dual-colour ELISpot assay. The presence of these cytokines after antigen specific stimulation will be indicative of the Th1 response. Additionally, we will assess T cell polyfunctionality and the induction of immunological memory and homing patterns by intracellular cytokine staining and surface staining by flow cytometry. KWTRP has a 17 colour flow cytometry allowing extensive phenotypic characterization.

Detailed epitope mapping studies are also planned again using flow cytometry to more fully examine the depth of vaccine induced T cell responses. Th1 proliferations will be assessed by Ann Jerse Uniformed Services University, Bethesda, Maryland, USA.

Antibody Responses

The induction of antibody will be measured by a standard endpoint ELISA assay using peptides covering recombinant protein antigens, purified antigens, and defined OMVs. The protein antigens will be generated during the fabrication of protein microarrays, and as candidate vaccines. Antigens will be coated onto microtiter plates, and circulating antibody responses in sera detected by incubating serial dilutions of sera with antigens; binding of total IgG, IgA, and IgG subclasses will be determined using specific secondary antibodies. Antibody titres from individuals approximately two weeks following the final immunisation and/or infected individuals will be compared with levels measured at study entry, and statistical significance analysed by unpaired T tests.

After the final immunization, PBMCs will be collected for isolation of antigen-specific memory B cells. B-cell sorting will require a blood draw of 50 ml (appendix A: schedule of procedures). The aim of this will be to generate human monoclonal antibodies (mAbs) against key vaccine candidates; these mAbs will be a valuable resource during the development and evaluation of vaccine candidates. Generated monoclonal antibodies will be characterized and testing for effector function. Specifically, in those participants where cross-reactive plasma will be identified in section 10.1, antigen-specific memory B cells will be isolated two weeks post second immunization.

The immunoglobulin genes will be independently amplified and the variable gene regions cloned into expression vectors. Monoclonal antibodies will then be expressed and purified. These monoclonal antibodies will be characterized and tested for their binding capacity against Ng antigens. This work will be done at KEMRI-CGMRC where similar studies are currently on going. We will select samples for isolating antigen-specific cells based on individuals' responses as judged by results using immune sera with protein microarrays and ELISA.

Sample storage:

PBMC, serum and plasma samples will be stored as indicated in the Laboratory Analytical Plan (LAP) at laboratories at KEMRI-WTRP- (Kilifi, Kenya), Universities of Oxford, and Manchester (UK), and Uniformed Services University (USA) and analysed for the purposes outlined within this protocol as listed above. Consent will be sought for long-term storage at KEMRI-WTRP in Kenya, any future usage of these samples will seek the necessary approvals from CGMRC and KEMRI-SERU. Samples not consented for long-term storage are to be stored until study close out and thereafter disposed of following the local country requirements and regulations relating to the disposal of biological research samples.

10.7. Drug concentration measurements

n/a

10.8. Health economic measures

n/a

11. ASSESSMENT OF SAFETY

11.1. Adverse Events (AEs)

This study is being conducted with a licensed product in other countries than Kenya (and administered using the same dose and regimen as in countries where it is licensed), and as such safety monitoring will therefore focus on detecting any suspected, unexpected, serious, adverse reactions, i.e. SUSARs. We will monitor for solicited adverse events.

11.2. Definitions and monitoring of AEs

Adverse event (AE)	Any untoward medical occurrence in a participant to whom a medicinal product has been administered, including occurrences which are not necessarily caused by or related to that product.
Adverse reaction (AR)	An untoward and unintended response in a participant to an investigational medicinal product which is related to any dose administered to that participant.<br><br>The phrase "response to an investigational medicinal product" means that a causal relationship between a trial medication and an AE is at least a reasonable possibility, i.e. the relationship cannot be ruled out.<br><br>All cases judged by either the reporting medically qualified professional or the Sponsor as having a reasonable suspected causal relationship to the trial medication qualify as adverse reactions.
Serious Adverse Event (SAE)	A serious adverse event is any untoward medical occurrence that:<br>• results in death<br>• is life-threatening<br>• requires inpatient hospitalisation or prolongation of existing hospitalisation<br>• results in persistent or significant disability/incapacity

	• consists of a congenital anomaly or birth defect.<br><br>Other 'important medical events' may also be considered serious if they jeopardise the participant or require an intervention to prevent one of the above consequences.<br><br>NOTE: The term "life-threatening" in the definition of "serious" refers to an event in which the participant was at risk of death at the time of the event; it does not refer to an event which hypothetically might have caused death if it were more severe.
Serious Adverse Reaction (SAR)	An adverse event that is both serious and, in the opinion of the reporting Investigator, believed with reasonable probability to be due to one of the trial treatments, based on the information provided.
Suspected Unexpected Serious Adverse Reaction (SUSAR)	A serious adverse reaction, the nature and severity of which is not consistent with the information about the medicinal product in question set out:<br>• in the case of a product with a marketing authorisation, in the summary of product characteristics (SmPC) for that product<br>• in the case of any other investigational medicinal product, in the investigator's brochure (IB) relating to the trial in question.

NB: to avoid confusion or misunderstanding of the difference between the terms "serious" and "severe", the following note of clarification is provided: "Severe" is often used to describe intensity of a specific event, which may be of relatively minor medical significance. "Seriousness" is the regulatory definition supplied above.

11.3. **Causality**

The relationship of relevant serious adverse events to the trial medication must be determined by a medically qualified individual according to the following definitions:

Related: The adverse event follows a reasonable temporal sequence from trial medication administration. It cannot reasonably be attributed to any other cause.

Not Related: The adverse event is probably produced by the participant's clinical state or by other modes of therapy administered to the participant.

For the purpose of expedited safety reporting, all possibly, probably or definitely related AEs are considered Investigational Medicinal Product-related AEs.

Details on AE definitions are provided below and in the SOM.

RELATIONSHIP	DESCRIPTION
Unrelated	There is no evidence of any causal relationship
Unlikely	There is little evidence to suggest that there is a causal relationship (for example, the event did not occur within a reasonable time after administration of the trial vaccination). There is another reasonable explanation for the event (for example, the patient's clinical condition, other concomitant treatment).
Possible	There is some evidence to suggest a causal relationship (for example, because the event occurs within a reasonable time after administration of the trial vaccination). However, the influence of other factors may have contributed to the event (for example, the patient's clinical condition, other concomitant treatments).
Probable	There is evidence to suggest a causal relationship and the influence of other factors is unlikely.
Definitely	There is clear evidence to suggest a causal relationship and other possible contributing factors can be ruled out.

11.4. Procedures for Recording Adverse Events

Documenting AEs

AEs are observed by the Investigator or reported by the volunteer, will be recorded on the CRF, whether or not attributed to trial vaccination.

The following information will be recorded: description, date of onset and end date, severity, whether or not it is a serious adverse event, assessment of relatedness to investigational medicinal products and whether treatment was required. Follow-up information should be provided as necessary.

Reporting Serious Adverse Events (SAEs) and/or Unexpected AEs

Should investigators become aware of a serious adverse event the following information will be recorded: description, date of onset and end date, severity, assessment of relatedness to trial medication, other suspect drug or device and action taken. Follow-up information should be provided as necessary.

The severity of events will be assessed on the following scale: 1 = mild, 2 = moderate, 3 = severe.

SAEs considered related to the trial medication as judged by a medically qualified investigator or the Sponsor will be followed either until resolution, or the event is considered stable.

It will be left to the Investigator's clinical judgment to decide whether or not an AE is of sufficient severity to prevent the participant receiving a second dose of vaccine. A participant may also voluntarily withdraw from treatment due to what he or she perceives as an intolerable AE. If either of these occurs, the participant will be given appropriate care under medical supervision until symptoms cease, or the condition becomes stable, and may participate in immunogenicity assessments according to schedule.

Reporting of SAEs

The period of recording SAEs will be from the time of taking informed consent to the last study visit for that participant.

All SAEs must be reported on the SAE reporting form to SERU within 24 hours of the Site Study Team becoming aware of the event, with the site Principal Investigator being included in this correspondence.

All SAE information must be recorded on an SAE form and faxed, or scanned and emailed, to SERU. Additional and further requested information (follow-up or corrections to the original case) will be detailed on a new SAE Report Form and faxed/emailed to SERU.

All SUSARs will be reported by the Principal investigator to the relevant Regulatory Authority and to the Ethics Committees and other parties as applicable. For fatal and life-threatening SUSARS, this

will be done no later than seven calendar days after the Sponsor or delegate is first aware of the reaction or following timelines required in the individual countries, whichever one is more stringent. Any additional relevant information will be reported within 8 calendar days of the initial report. All other SUSARs will be reported within 15 calendar days.

DSMB

An independent Data Safety and Monitoring Board (DSMB) will be set up consisting of qualified volunteers with the necessary knowledge of clinical trials. The DSMB will receive summary reports, prior to each meeting. All data reviewed by the DSMB will be in the strictest confidence. A DSMB charter will outline its responsibilities, number of interim reports and how it will operate. Interim reports will be prepared by the Trial data manager.

All DSMB recommendations will be communicated to the PI. The PI will be responsible for submitting the written DSMB summary reports with recommendations as applicable to local/ national ethics committees and other applicable groups.

11.5. Emergency Procedures

The KEMRI clinic in Malindihas an emergency trolley and staff is trained to offer emergency procedures should anaphylactic shock arise following vaccination.

11.3. Pregnancy

Any pregnancy which occurs during the clinical trial, and the outcome of the pregnancy, should be recorded and followed up for congenital abnormality or birth defect, at which point it would fall within the definition of “serious”.

Procedures for reporting any protocol violation(s). Protocol violation (s) will be reported promptly to the Ethics Committee and Regulatory Authority.

12. STATISTICS

12.1. Determination of sample size

The sample size is based on our experience of evaluating the humoral responses to Bexsero® in 50 healthy individuals from the UK with a low likelihood of previous exposure to Ng. In that study, we would not expect the vaccine to elicit memory responses, and considered the previous group as naïve. We assessed immune responses using protein microarrays which contain over 80 purified recombinant antigens that are present in the OMVs from the vaccine. This allowed us to detect IgG responses against a diverse array of antigens. We identified six antigens using the Nm microarrays which exhibited significant enhancement following vaccination in sera from these volunteers.

In the present study, we plan to enroll around twice the number of subjects from our cohort of sex workers who have high rates of exposure to Ng, and so are more likely to mount a serological and/or cellular response to the vaccine than the general population. This should provide sufficient lead candidate antigens for vaccine development, including recombinant proteins and/or engineering OMVs.

12.2. Statistical and analytical plans

We will use protein microarrays to identify antigens in *Neisseria gonorrhoeae* which are recognised by antibodies elicited by Bexsero. We have pioneered the development and use of protein microarrays, which enable the detection of cross-reactive antibody responses against multiple antigens in parallel. We have generated protein microarrays consisting of membrane/surface proteins of *N. meningitidis*, and evaluated responses in 50 healthy volunteers immunised with an OMV–based vaccine. [33]. More than 80% of participants displayed significant serological responses detected by the microarrays, with around 10 antigens identified by responses in most individuals. We predict that we will observe similar responses to Bexsero (which contains an OMV) in our study population which is highly exposed to Ng infection so might be immunologically primed, with a high degree of sequence conservation (>90% identify) between Ng and meningococcal antigens. Therefore, we have chosen to use a similar number of volunteers for the current study as in our previous work.

13. Access to Data

Direct access will be granted to authorised representatives from the Sponsor, host institution and the regulatory authorities to permit trial-related monitoring, audits and inspections.

14. Data Recording and Record Keeping

Source documents are the documents where data are first recorded, and from which volunteers' CRF data are obtained. These include, but are not limited to, hospital records (from which medical history and previous and concurrent medication may be summarised into the CRF), clinical charts, and laboratory and pharmacy records.

CRF entries will be considered source data if the CRF is the site of the original recording (e.g. there is no other written or electronic record of data). All documents will be stored safely in confidential conditions. On all trial-specific documents, other than the signed consent, the volunteer will be referred to by the trial volunteer number/code, not by name.

All trial data will be entered in the clinical trial database, which are maintained through Standard Operating Procedures.

The volunteer will be identified by a unique trial specific number and/or code in any database. The name and any other identifying detail will NOT be included in any trial data electronic file.

15. Data entry at study site

The data collected at the site will be recorded onto the source documents by the study staff and entered into the clinical trial database.

15.1.1. Planned statistical methods

A detailed statistical analysis plan will be developed by prior to database lock and final analysis**.**

Demographics and baseline characteristics. We will collect demographics and baseline characteristics from existing databases (as participants derive from well-characterized cohort studies), including number of sexual partners in the past month, sexual role taking, sex work, and detection of any prior Gc infection, or Gc infection during the study. These data will be captured in the two existing KEMRI protocols in which participants will remain enrolled.

For the primary analysis, we will compare the difference in total IgG sero-reactivity between human pre-vaccination (week 0) and post-vaccination sera (week 10 and week 24). To determine the increase

in IgG reactivity for a particular antigen at week 10 or week 24, the value obtained from serum collected before immunization will be subtracted from the same value obtained from serum after immunization at week 10 or week 24. A two-tailed T-test will be used to calculate p-values and test for significance between doses per protein.

For the ELISPOT analysis, we will determine the proportion of IFNg secreting T cells per 1 million PBMCs in samples tested 10 weeks post vaccination (week 10). To account for background responses, spot forming units (SFUs) detected at baseline (week 0) samples prior to vaccination, and the average SFUs identified in wells with mock peptides (negative control; at week 0) will be subtracted from responses at week 10. The proportions antigen-specific IFNg secreting T cells will be compared using non-parametric tests between the different peptide pools used for stimulation, between vaccinees to identify the peptide pool yielding highest activation (pre-vaccination (week 0) and post-vaccination sera (week 10)), and between vaccinees with the highest T cell responses.

Missing data. There will be no imputation of missing data.

15.1.1.1. Efficacy population n/a

15.1.1.2. Safety population n/a

15.1.1.3. Interim analysis n/a

15.1.1.4. Efficacy analyses n/a

15.1.1.5. Safety analyses n/a

15.1.1.6. Amendments to statistical analysis n/a

15.1.1.7. **Stopping criteria for termination of trial** n/a

16. DIRECT ACCESS TO SOURCE DATA/DOCUMENTS

Direct access will be granted to authorised representatives from the Sponsor, host institution and the regulatory authorities to permit trial-related monitoring, audits and inspections. Ultimately any gonococcal genome sequence data deriving from any Ng isolates, without participant identifying information, will be made available publicly.

17. QUALITY CONTROL AND QUALITY ASSURANCE

To ensure the quality and reliability of the data collected and generated and the ethical conduct of this study, a Study Operations Manual will be developed. All deviations will be reported and investigated. The Study Operations Manual describes reporting and deviation documentation requirements and procedures. Regular monitoring will be performed according to ICH-GCP as indicated in Section 17.2. An independent audit of the study and study sites may be performed by the Sponsor or designee to establish the status of applicable quality systems. Inspection by regulatory authorities may also occur. By signing the protocol, the Principal Investigators agree to facilitate study related monitoring, audits, IEC/IRB review and regulatory inspection(s) and direct access to source documents. Such information will be treated as strictly confidential and under no circumstances be made publicly available.

17.1. **Choice of investigators.** Investigators in Kenya have extensive experience with key population cohort studies (from which participants for the Gc vaccine study will derive), as well as immunogenicity testing of vaccines at the KWTRP lab's in Kilifi. Collaborators in the UK (Chris Tang and Jeremy Derrick), and USA (Ann Jerse) are leading researchers in the Gc-field, and have extensive experience in the design, development, and post-implementation analysis of vaccine against the closely-related pathogen, *N. meningitidis*.

17.2. Monitors and monitoring plan

Study monitoring will be conducted by the KWTRP. Monitoring will be conducted to ensure that: the rights and wellbeing of volunteers are protected; the reported data are accurate, complete and verifiable from source documents; and that study conduct complies with the currently approved protocol, standard operating procedures, Good Clinical Practice (GCP) and other applicable regulatory requirements.

Study monitors will visit the study sites to review all trial documents including volunteer screening and enrolment logs, informed consent forms, source documents, CRFs, laboratory

and medical records. The specific objectives of a monitoring visit will be 1). to verify the existence of adequately signed informed consent forms for each enrolled volunteer; 2) to verify the prompt, complete and accurate recording of data, and prompt reporting of all SAEs and SUSARs; 3) to verify the quality and accuracy of data by validation of CRFs against the source documents such as volunteers' medical records, laboratory reports, and any other relevant original data; 4) to verify adequate IMP supply, storage, management, and accountability; and 5) to ensure protection of study volunteers, and investigators' compliance with the protocol, regulatory requirements and applicable guidelines.

Study investigators and volunteers agree that the study monitor may review study facilities and source records, and observe the performance of study procedures. Additionally, study investigators will permit inspection or audit of the study facilities and all study-related records by relevant regulatory authorities, SERU, and/or representatives of the Sponsor. All information collected during monitoring or audit visits will be treated as strictly confidential and will under no circumstances be made publicly available.

18. INTELLECTUAL PROPERTY

Any intellectual property rights that arise from the work will be safeguarded according to the current KEMRI guidelines and the Industrial Property Act of 2001, sections 32, 58 and 80. The scientific and intellectual contributions of all persons involved in the research will be appropriately acknowledged in all publications and presentations arising from the work.

19. TIME FRAME/DURATION OF THE TRIAL

Activity	Q3-4, 2019	Q1-2, 2020	Q3-4, 2020	2021-22
**Consortium meeting**	✓			
**Cohort preparations**	✓	✓	✓	
**Clinical Trial**			✓ 1	✓
**Clinical Immunology**			✓	✓
**Dissemination & communication**			✓	✓

1). Screening may start in December 2020

20. ETHICS

Ethical approval will be sought from KEMRI Scientific & Ethics Review Unit (SERU), and the Oxford Tropical Research Ethics Committee (OXTREC).

20.1. Human Subjects

The study involves human subjects and the principles below will be observed.

“First, do no harm.”

The study staff will make every effort to protect participants’ privacy and confidentiality. However, it is possible that others may learn a participant is taking part in a study and make assumptions about his health or sexuality. All participants are known by a number, we will not include names or other identifying information on participants’ records. All study documents will be stored on a password-protected computer only accessible by authorized staff.

Participation in this study is voluntary. Participants are free to decide if they want to take part or not, which they will be informed about before obtaining consent. If participants do agree to participate they can change their mind at any time without any consequences.

Participants will be recruited from four KEMRI cohorts studies (SERU No. 894, 3520, 3778 and 1224) in Mtwapa or Malindi. The KEMRI cohort studies follow both HIV uninfected and infected individuals at-risk for Ng infection, including male and female sex workers, and MSM. For this study, we will screen participants aged 18-25 years. At present, over 100 HIV-negative participants (100 MSM, 5 FSW), and 29 HIV-positive participants (25 MSM and 4 FSW) aged 18-25 years are eligible for screening.

Prospective participants will be invited to attend an individual information session in which the purpose of the Gc vaccine study will be discussed, and questions can be asked.
We will ensure that participants understand all aspects of the study, including which samples will be collected (i.e. blood, and throat, vaginal/rectal swab samples, and urine) and what volumes will be collected.

We will explain that the aim of the study is to assess how the immune system responds to a licensed Meningococcal B vaccine (Bexsero®). Over 17 million doses of the vaccine have been given worldwide, but the vaccine is not licensed in Kenya (as meningococcal B infection is not common). Meningococcal B vaccine (Bexsero®) prevents only against the meningococcal B strain which is common in Europe, Australia, and the Unites States.

Due to genetic similarities between the two organisms causing gonorrhoea and meningococcal B disease, the vaccination with Bexsero® may be effective against gonorrhoea.

The KEMRI cohort studies in Mtwapa and Malindi are supported by a ‘key populations study’ community advisory board (CAB). The CAB consists of stakeholders from LGBTQ-organizations, business leaders, bar owners, religious leaders, community security, chiefs,

police, village elders, as well as lawyers, human -rights activists, and leaders from several LGBTQ CBOs along the Kenyan coast.

The CAB is an integral part of KEMRI's HIV and STI engagements; building and fostering partnerships between researchers and local study communities, helping to strengthen local trust and mutual understanding of research issues; and ensuring that values and cultural differences among participants are respected. KEMRI organises quarterly engagement meetings with the CAB during which planned studies will be shared. The CAB will be informed about the Gc-vaccine study, and we will ensure that CAB members can ask questions about study. We will also inform Kilifi County Health Department, Malindi stake holder's forum, and members of the Kilifi Community Representatives in ongoing quarterly engagements.

Prospective participants who consider participation in the Gc vaccine study may want to consult CAB members. The CAB will also be informed about study progress, and when summary data will become available from the study.

20.2. Community Considerations

While the study does not offer direct benefits (i.e. participants will receive the same research services as in the KEMRI cohorts that they participate in already), researchers will get new insights from immune responses to the Meningococcal B vaccine (Bexsero®) and if any component of that vaccine can be improved upon in the future to make a better vaccine against gonorrhoea.

While the immunological analysis of the study will likely take up to two years before results can be disseminated, our community dissemination plan will include updates on study start and completion for participants, and share information with community stakeholders on any

further information concerning 'Bexsero vaccination studies' that may be conducted elsewhere.

20.3. Informed Consent

Written informed consent will be obtained from all study participants by the research counsellor. Informed consent document (ICD) will be available in English and Kiswahili. During screening, we will provide information to prospective participants and summarise key sections of the ICD. We will ensure sufficient time to provide information and allow for questions. Counsellors and clinicians will adhere to standard operating procedures in obtaining consent**.**

20.4. Compensation

The Meningococcal B vaccine (Bexsero®), and study related tests and visits will be free of charge. Participants will be given Ksh500 at the screening visit, and Ksh1000 at the 4 follow up visits to cover the cost of transportation. The Ksh1000 per study visit for participation in a vaccine study is similar to a previous vaccine study (Yellow Fever fractional doses) conducted at the KEMRI clinic in Malindi during 2019-20, and to a planned HIV vaccine study to be conducted in 2020. Participants who will travel from Mtwapa to Malindi will receive additional travel compensation of public transport costs.

20.5. Patient Data Protection/Confidentiality

Study information will be kept confidential by storing paper copies in locked cabinets and electronic copies in password-protected computers that will be only accessible to study staff. Identifying information will be destroyed after study completion according to KEMRI policy.

20.6. Data Sharing

The knowledge gained from this research will be shared and published in summary form, without revealing identity of participants. In future, information collected during this study may be used to support new research by other researchers. In all cases, we will only share information with other researchers in ways that do not reveal identity of participants. Any future research must first be approved by a county or national expert committee to make sure that the interests of participants and their communities are protected.

Publications from this study are required to comply with the Wellcome Trust Open Access policy: https://wellcome.ac.uk/funding/guidance/guidance-wellcome-trust-centres-and-major-overseas-programmes, and the policy on data, software and materials management and sharing: https://wellcome.ac.uk/funding/guidance/policy-data-software-materials-management-and-sharing.

Upon completion of the study, participants will be invited for a study findings dissemination session. We will also share study findings with the CAB.

20.7. Safety

Upon completion of the Gonoccocal vaccine study, participants may continue in the KEMRI cohorts at the KEMRI clinic in Mtwapa or Malindi as appropriate.

20.8. Material Transfer Agreement (if applicable)

The study will store samples at the KWTRP laboratories in Kilifi, and share a portion of the samples with collaborators in the UK, and USA. Material transfer agreements with collaborators in the UK, and USA will be made where appropriate, and specify shipment of samples as described in the methods.

21. ARCHIVING AND RECORD RETENTION

Electronic data bases and source documents will be stored for a minimum of 10 years and follow programme policies on archiving and data storage.

22. FINANCING AND INSURANCE

The study will be funded primarily by a collaborative grant from the Wellcome Trust.

22.1. Budget

Item	US$	Ksh
Staff	176000	17,600000
Laboratory assays and cohort support	32750[1]	3,275000[1]
Bexsero Vaccine, GSK, UK	12000	1,200000
Immunology assays, including shipment	39975	3,972500
Meeting attendance	5000	500000
Total	265,725	26,572500

1). Inclusive of up to 15 viral load assays.

Budget justification

Personnel. Personnel includes support for a clinical officer, pharmacy technologist, counsellors, data staff, community engagement staff, field workers, and house-keeping staff.

Laboratory assays, **cohort support, and clinic visits** at the KEMRI clinic include X-pert CT/NG testing (US$ $ 22.5 per assay; 3 anatomical sites per visit; 3 visits). Cohort support includes community engagement activities, tracing, information session and community advisory board training. **Clinic visits** include a screening visit (Ks500) and 4 follow up visits (1000Ksh per visit).

Meningococcal B vaccine (Bexsero®) @ 120US$ per dose (2 doses, 50 participants).

Immunological assays. Fresh T-cell Elispot, possible B-cell cloning and cell sorting on site; shipment of cells, and serum to Oxford, Manchester (UK), and Bethesda, USA.

22.2. Insurance

The University of Oxford has a specialist insurance policy in place which would operate in the event of any volunteer suffering harm as a result of their involvement in the research (Newline Underwriting Management Ltd, at Lloyd's of London).

Indemnity

KWTRP have professional indemnity cover for KEMRI staff.

If any volunteer is harmed as a result of this trial, medical care will be provided. The sponsor will provide care until complete cure or stabilization of a research related injury. The injured research volunteer will be given the best care available within the country for the research related injury. Research volunteers shall not be required to waive their legal rights for redress in courts of law.

Negligent Harm

Indemnity and/or compensation for negligent harm arising specifically from an accidental injury for which the University is legally liable as the Research Sponsor will be covered by the University of Oxford.

Non-Negligent Harm

Indemnity and/or compensation for harm arising specifically from an accidental injury, and occurring as a consequence of the Research volunteers' participation in the trial for which the University is the Research Sponsor will be covered by the University of Oxford.

23. TRIAL MANAGEMENT

The trial will be registered through ClinicalTrials.gov. The Principal Investigator will be responsible for all aspects of the study at the study site.

Study Supervision

The Principal Investigator will work closely with his/her study team to implement the study, address issues in a timely manner, assure consistent documentation, and compile and provide study progress reports to the medical monitor. Accrual and retention rates, safety of study volunteers, and other relevant parameters will be closely monitored by the study team, Principal Investigator, and Medical Monitor.

Investigator's Records

Study records include administrative documentation, including reports and correspondence relating to the study, as well as documentation related to each volunteer screened for and/or enrolled in the study (e.g., screening and enrolment logs, locator information forms, informed consent forms, laboratory reports, case report forms, and all other source documents). The Principal Investigator will maintain and store, in a secure manner, complete, accurate, and current study records for a minimum of 2 years.

24. REPORTING, DISSEMINATION AND NOTIFICATION OF RESULTS

An initial descriptive manuscript on key population participation in a Bexsero trial in Kenya will be prepared promptly after data analysis is completed using mutually accepted Publication Guidelines. A primary manuscript assessing if immunisation of individuals at risk for gonococcal infection with 4CMenB (Bexsero) will elicit humoral and T cell cross-reactive responses against Ng immune responses will be prepared when all available data is analysed. Authorship criteria will be based on contributions to the design, work, analysis and writing of the study report. A summary of the

main findings will be developed that will be in non-technical language and shared with stake holders, and the CAB.

25. APPENDICES

1. Appendix A
2. CV of Prof Chris Tang
3. Ethics certificates of investigators

25.1. Roles of Investigators

Chris Tang	Chief investigator Gonococcal Vaccine Initiative, study design
Eduard Sanders	clinical trial principal investigator, study design, study oversight Bexsero trial, medically qualified and on-site
Philip Bejon	Protocol development, study design, immunology study oversight
Clara Agutu	Protocol development, clinical trial investigator, medically qualified and on-site
Eunice Nduati	B-cell immunologist
Susan Graham	Protocol development, clinical trial investigator

REFERENCES

1. DALYs GBD, Collaborators H, Murray CJ, Barber RM, Foreman KJ, Abbasoglu Ozgoren A, et al. **Global, regional, and national disability-adjusted life years (DALYs) for 306 diseases and injuries and healthy life expectancy (HALE) for 188 countries, 1990-2013: quantifying the epidemiological transition**. *Lancet* 2015; 386(10009):2145-2191.
2. Brunham RC, Gottlieb SL, Paavonen J. **Pelvic inflammatory disease**. *N Engl J Med* 2015; 372(21):2039-2048.
3. Vincent LR, Jerse AE. **Biological feasibility and importance of a gonorrhea vaccine for global public health**. *Vaccine* 2018.
4. Mlisana K, Naicker N, Werner L, Roberts L, van Loggerenberg F, Baxter C, et al. **Symptomatic vaginal discharge is a poor predictor of sexually transmitted infections and genital tract inflammation in high-risk women in South Africa**. *J Infect Dis* 2012; 206(1):6-14.
5. Rowley J, Vander Hoorn S, Korenromp E, Low N, Unemo M, Abu-Raddad LJ, et al. **Chlamydia, gonorrhoea, trichomoniasis and syphilis: global prevalence and incidence estimates, 2016**. *Bulletin of the World Health Organization* 2019; 97(8):548-562P.
6. Unemo M, Bradshaw CS, Hocking JS, de Vries HJC, Francis SC, Mabey D, et al. **Sexually transmitted infections: challenges ahead**. *Lancet Infect Dis* 2017; 17(8):e235-e279.
7. Wi T, Lahra MM, Ndowa F, Bala M, Dillon JR, Ramon-Pardo P, et al. **Antimicrobial resistance in Neisseria gonorrhoeae: Global surveillance and a call for international collaborative action**. *PLoS Med* 2017; 14(7):e1002344.
8. Whitney CG, Farley MM, Hadler J, Harrison LH, Bennett NM, Lynfield R, et al. **Decline in invasive pneumococcal disease after the introduction of protein-polysaccharide conjugate vaccine**. *N Engl J Med* 2003; 348(18):1737-1746.
9. Jerse AE, Bash MC, Russell MW. **Vaccines against gonorrhea: current status and future challenges**. *Vaccine* 2014; 32(14):1579-1587.

10. Liu Y, Islam EA, Jarvis GA, Gray-Owen SD, Russell MW. **Neisseria gonorrhoeae selectively suppresses the development of Th1 and Th2 cells, and enhances Th17 cell responses, through TGF-beta-dependent mechanisms**. *Mucosal immunology* 2012; 5(3):320-331.
11. Liu Y, Liu W, Russell MW. **Suppression of host adaptive immune responses by Neisseria gonorrhoeae: role of interleukin 10 and type 1 regulatory T cells**. *Mucosal immunology* 2014; 7(1):165-176.
12. Greenberg L, Diena BB, Ashton FA, Wallace R, Kenny CP, Znamirowski R, et al. **Gonococcal vaccine studies in Inuvik**. *Can J Public Health* 1974; 65(1):29-33.
13. Boslego JW, Tramont EC, Chung RC, McChesney DG, Ciak J, Sadoff JC, et al. **Efficacy trial of a parenteral gonococcal pilus vaccine in men**. *Vaccine* 1991; 9(3):154-162.
14. Ram S, McQuillen DP, Gulati S, Elkins C, Pangburn MK, Rice PA. **Binding of complement factor H to loop 5 of porin protein 1A: a molecular mechanism of serum resistance of nonsialylated Neisseria gonorrhoeae**. *The Journal of experimental medicine* 1998; 188(4):671-680.
15. Schneider MC, Prosser BE, Caesar JJ, Kugelberg E, Li S, Zhang Q, et al. **Neisseria meningitidis recruits factor H using protein mimicry of host carbohydrates**. *Nature* 2009; 458(7240):890-893.
16. Lewis LA, Gulati S, Burrowes E, Zheng B, Ram S, Rice PA. **alpha-2,3-sialyltransferase expression level impacts the kinetics of lipooligosaccharide sialylation, complement resistance, and the ability of Neisseria gonorrhoeae to colonize the murine genital tract**. *MBio* 2015; 6(1).
17. Ram S, Cullinane M, Blom AM, Gulati S, McQuillen DP, Monks BG, et al. **Binding of C4b-binding protein to porin: a molecular mechanism of serum resistance of Neisseria gonorrhoeae**. *The Journal of experimental medicine* 2001; 193(3):281-295.
18. Deo P, Chow SH, Hay ID, Kleifeld O, Costin A, Elgass KD, et al. **Outer membrane vesicles from Neisseria gonorrhoeae target PorB to mitochondria and induce apoptosis**. *PLoS Pathog* 2018; 14(3):e1006945.
19. Plummer FA, Chubb H, Simonsen JN, Bosire M, Slaney L, Maclean I, et al. **Antibody to Rmp (outer membrane protein 3) increases susceptibility to gonococcal infection**. *The Journal of clinical investigation* 1993; 91(1):339-343.
20. Rice PA, Vayo HE, Tam MR, Blake MS. **Immunoglobulin G antibodies directed against protein III block killing of serum-resistant Neisseria gonorrhoeae by immune serum**. *The Journal of experimental medicine* 1986; 164(5):1735-1748.
21. Feavers IM, Maiden MCJ. **Recent Progress in the Prevention of Serogroup B Meningococcal Disease**. *Clinical and vaccine immunology : CVI* 2017; 24(5).
22. Petousis-Harris H, Paynter J, Morgan J, Saxton P, McArdle B, Goodyear-Smith F, et al. **Effectiveness of a group B outer membrane vesicle meningococcal vaccine against gonorrhoea in New Zealand: a retrospective case-control study**. *Lancet* 2017; 390(10102):1603-1610.
23. Ladhani SN, Campbell H, Parikh SR, Saliba V, Borrow R, Ramsay M. **The introduction of the meningococcal B (MenB) vaccine (Bexsero(R)) into the national infant immunisation programme--New challenges for public health**. *J Infect* 2015; 71(6):611-614.
24. Plummer FA, Simonsen JN, Chubb H, Slaney L, Kimata J, Bosire M, et al. **Epidemiologic evidence for the development of serovar-specific immunity after gonococcal infection**. *The Journal of clinical investigation* 1989; 83(5):1472-1476.
25. Semchenko EA, Tan A, Borrow R, Seib KL. **The serogroup B meningococcal vaccine Bexsero elicits antibodies to Neisseria gonorrhoeae**. *Clin Infect Dis* 2018.
26. Perrett KP, McVernon J, Richmond PC, Marshall H, Nissen M, August A, et al. **Immune responses to a recombinant, four-component, meningococcal serogroup B vaccine (4CMenB) in adolescents: a phase III, randomized, multicentre, lot-to-lot consistency study**. *Vaccine* 2015; 33(39):5217-5224.
27. McNeil MM, Weintraub ES, Duffy J, Sukumaran L, Jacobsen SJ, Klein NP, et al. **Risk of anaphylaxis after vaccination in children and adults**. *J Allergy Clin Immunol* 2016; 137(3):868-878.
28. Rollier CS, Dold C, Marsay L, Sadarangani M, Pollard AJ. **The capsular group B meningococcal vaccine, 4CMenB : clinical experience and potential efficacy**. *Expert Opin Biol Ther* 2015; 15(1):131-142.
29. Oviedo-Orta E, Ahmed S, Rappuoli R, Black S. **Prevention and control of meningococcal outbreaks: The emerging role of serogroup B meningococcal vaccines**. *Vaccine* 2015; 33(31):3628-3635.
30. Newman L, Rowley J, Vander Hoorn S, Wijesooriya NS, Unemo M, Low N, et al. **Global Estimates of the Prevalence and Incidence of Four Curable Sexually Transmitted Infections in 2012 Based on Systematic Review and Global Reporting**. *PLoS One* 2015; 10(12):e0143304.
31. Galvin SR, Cohen MS. **The role of sexually transmitted diseases in HIV transmission**. *Nat Rev Microbiol* 2004; 2(1):33-42.
32. Wi T, Lahra MM, Ndowa F, Bala M, Dillon JR, Ramon-Pardo P, et al. **Antimicrobial resistance in Neisseria gonorrhoeae: Global surveillance and a call for international collaborative action**. *PLoS Med* 2017; 14(7):e1002344.

33. Awanye AM, Chang CM, Wheeler JX, Chan H, Marsay L, Dold C, et al. **Immunogenicity profiling of protein antigens from capsular group B Neisseria meningitidis**. *Scientific reports* 2019; 9(1):6843.
