# Supplementary material for: Profiling IgG and IgA antibody responses during vaccination and infection in a high-risk gonorrhoea population
Source: Nat Commun. 2024 Aug 7;15:6712. doi: 10.1038/s41467-024-51053-x (PMC11306574; doi:10.1038/s41467-024-51053-x)
Supplement: Supplementary file 3 — Description of Additional Supplementary Files [file 41467_2024_51053_MOESM3_ESM.pdf]

<u>Description of Additional Supplementary Files</u>

Supplementary Data 1: Table of antigens used for the microarray. Antigens are listed by common name, and NGO and NEIS designation numbers.
