# Supplementary material for: Profiling IgG and IgA antibody responses during vaccination and infection in a high-risk gonorrhoea population
Source: Nat Commun. 2024 Aug 7;15:6712. doi: 10.1038/s41467-024-51053-x (PMC11306574; doi:10.1038/s41467-024-51053-x)
Supplement: Supplementary file 5 — Reporting Summary [file 41467_2024_51053_MOESM5_ESM.pdf]

nature portfolio

Corresponding author(s): Jeremy Derrick; Ed Sanders; Chris Tang

Last updated by author(s): Dec 8, 2023

Reporting Summary

Nature Portfolio wishes to improve the reproducibility of the work that we publish. This form provides structure for consistency and transparency in reporting. For further information on Nature Portfolio policies, see our Editorial Policies and the Editorial Policy Checklist.

Statistics

For all statistical analyses, confirm that the following items are present in the figure legend, table legend, main text, or Methods section.

n/a	Confirmed	
☐	☒	The exact sample size (*n*) for each experimental group/condition, given as a discrete number and unit of measurement
☐	☒	A statement on whether measurements were taken from distinct samples or whether the same sample was measured repeatedly
☐	☒	The statistical test(s) used AND whether they are one- or two-sided<br>*Only common tests should be described solely by name; describe more complex techniques in the Methods section.*
☐	☒	A description of all covariates tested
☐	☒	A description of any assumptions or corrections, such as tests of normality and adjustment for multiple comparisons
☐	☒	A full description of the statistical parameters including central tendency (e.g. means) or other basic estimates (e.g. regression coefficient) AND variation (e.g. standard deviation) or associated estimates of uncertainty (e.g. confidence intervals)
☐	☒	For null hypothesis testing, the test statistic (e.g. *F*, *t*, *r*) with confidence intervals, effect sizes, degrees of freedom and *P* value noted<br>*Give P values as exact values whenever suitable.*
☒	☐	For Bayesian analysis, information on the choice of priors and Markov chain Monte Carlo settings
☒	☐	For hierarchical and complex designs, identification of the appropriate level for tests and full reporting of outcomes
☒	☐	Estimates of effect sizes (e.g. Cohen's *d*, Pearson's *r*), indicating how they were calculated

Our web collection on statistics for biologists contains articles on many of the points above.

Software and code

Policy information about availability of computer code

Data collection	Microarray spot intensities were quantified using Mapix 9.1.0 Microarray Image Analysis software (Innopsys).
Data analysis	Data were analysed using dedicated applications in R (version 4.2.3); asca was implemented from multiblock version 0.8.7; tSNE was implemented from Rtsne version 0.17; statistical analysis sued the Wilcoxon Rank Sum and Signed Rank Tests package (version 3.6.2). PCA analysis used the prcomp and factoextra packages version 1.0.7, implemented in R version 4.3.3.

For manuscripts utilizing custom algorithms or software that are central to the research but not yet described in published literature, software must be made available to editors and reviewers. We strongly encourage code deposition in a community repository (e.g. GitHub). See the Nature Portfolio guidelines for submitting code & software for further information.

Data

Policy information about availability of data

All manuscripts must include a data availability statement. This statement should provide the following information, where applicable:

- Accession codes, unique identifiers, or web links for publicly available datasets
- A description of any restrictions on data availability
- For clinical datasets or third party data, please ensure that the statement adheres to our policy

The microarray data generated in this study have been deposited in the GEO (Gene Expression Omnibus) database under accession code GSE269648 [https://www.ncbi.nlm.nih.gov/geo/].

Research involving human participants, their data, or biological material

Policy information about studies with human participants or human data. See also policy information about sex, gender (identity/presentation), and sexual orientation and race, ethnicity and racism.

Reporting on sex and gender	We have analysed the potential influence of sex within the dataset but see no effect (Figs S5 and S6;)- this is noted in the test of the Results section (line 285-288). Even if an effect were to be observed, its interpretation would need to be qualified. First, the dataset is unbalanced, with the majority of participants being male. The small numbers of female participants make the statistical analysis weak. Second, the group comprises sex workers and MSM; male/female categories are subject to multiple potential confounding variables, which could lead to misleading results. We therefore draw no conclusions in this study from sex or gender effects, although this would be an important area to investigate in further work.
Reporting on race, ethnicity, or other socially relevant groupings	The material studied was derived from a community in costal Kenya who are at high risk of gonorrhoea. In this study we do not, however, present data which makes any comparison with other racial groups- that, again, would need to be the subject of a separate study.
Population characteristics	These are described in the relevant sections of Methods. In addition to the details given above, participants were aged between 18 and 25 years old for the vaccine study and 18 and 49 years for the cohort study.
Recruitment	Participants included sex workers and MSM who were attended to at the Kenyan Medical Research Institute clinics in Mtwapa, and Malindi. Participants derived from ongoing cohort studies were established to estimate HIV incidence or assess uptake of care when participants were diagnosed with HIV. Cohort participants were mobilised through peer referral, or at bars, night clubs and other places where sex work was negotiated. The Ethical approval for NG isolate and serum shipment to the University of Oxford was granted by the Kenya Medical Research Institute (KEMRI) Scientific and Ethical Review Unit (approval number: 2842).
Ethics oversight	The vaccine trial was approved by the KEMRI Scientific and Ethical Review Unit (approval number: CSC 182) and the University of Oxford (approval number: 16-20). Ethical approval for the analysis of gonococcal isolates (the historical cohort) and serum was granted by the KEMRI Scientific and Ethical Review Unit (approval number: 2842).

Note that full information on the approval of the study protocol must also be provided in the manuscript.

Field-specific reporting

Please select the one below that is the best fit for your research. If you are not sure, read the appropriate sections before making your selection.

☒ Life sciences ☐ Behavioural & social sciences ☐ Ecological, evolutionary & environmental sciences

For a reference copy of the document with all sections, see nature.com/documents/nr-reporting-summary-flat.pdf

Life sciences study design

All studies must disclose on these points even when the disclosure is negative.

Sample size	Sample size for the study was guided by our previous experience with a similar study which we conducted using a meningococcal microarray (Awanye et al Sci Rep 9, Article number: 6843 (2019). The sample size in this study is about twice the number of participants: this was motivated by the different nature of the study, which was looking for cross-reactive antigens and may therefore have been less sensitive. Technical improvements optimised the sensitivity of this study: over 20 unique antigens were identified in the vaccine study using a stringent p-value cutoff of 0.01 (Tables 1 and 2). Numbers for the historical cohort study were roughly equivalent, allowing a direct comparison between the two groups. Despite the variation in the latter group, some separation by disease status was possible (Fig 7).
Data exclusions	No data were excluded from the analyses presented.
Replication	Each array contained a series of antibody concentrations, which were used as a standard to ensure reproducibility between slides and verify linearity. In addition, the reproducibility of the antigen profiling data was directly tested on a randomly chosen subset of serum samples for all the proteins contained in the microarray. Correlation R coefficients were obtained with values between 0.7 and 0.8.
Randomization	The vaccine and cohort studies did not require randomization- material from subjects was automatically assigned to pre- and post-vaccination groups, or before/during/after disease states.
Blinding	Laboratory workers engaged in microarray data collection were blinded to sample identities.

Reporting for specific materials, systems and methods

We require information from authors about some types of materials, experimental systems and methods used in many studies. Here, indicate whether each material, system or method listed is relevant to your study. If you are not sure if a list item applies to your research, read the appropriate section before selecting a response.

Materials & experimental systems

n/a	Involved in the study
☐	☒ Antibodies
☒	☐ Eukaryotic cell lines
☒	☐ Palaeontology and archaeology
☒	☐ Animals and other organisms
☐	☒ Clinical data
☒	☐ Dual use research of concern
☒	☐ Plants

Methods

n/a	Involved in the study
☒	☐ ChIP-seq
☒	☐ Flow cytometry
☒	☐ MRI-based neuroimaging

Antibodies

Antibodies used	For the immunogenicity probing, pre-adsorbed secondary antibodies were used. For IgG detection 100µl of Goat Anti-Human IgG Fc (DyLight® 650) secondary antibody (ab98622, Abcam, UK) was employed, using a working dilution of 1:5000 in blocking agent (SuperG™ Blocking Buffer (Grace Biolabs, US)). For IgA detection, the arrays were incubated with 100µl of Goat Anti-Human IgA alpha chain (DyLight® 650) secondary antibody (ab96998, Abcam, UK), using a working dilution of 1:5000 in blocking agent.
Validation	1. UK Goat Anti-Human IgG Fc (DyLight® 650) preadsorbed (ab98622, Abcam)<br>Host species: Goat; Target species: Human; certified by manufacturer for use in immunofluorescence.<br>2. Goat Anti-Human IgA alpha chain (DyLight® 650) secondary antibody (ab96998, Abcam, UK)<br>Host species: Goat; Target species: Human; certified by manufacturer for use in immunofluorescence; cross reactivity with other immunoglobulins and light chains is less than 0.1%.

Eukaryotic cell lines

Policy information about cell lines and Sex and Gender in Research

Cell line source(s)	*State the source of each cell line used and the sex of all primary cell lines and cells derived from human participants or vertebrate models.*
Authentication	*Describe the authentication procedures for each cell line used OR declare that none of the cell lines used were authenticated.*
Mycoplasma contamination	*Confirm that all cell lines tested negative for mycoplasma contamination OR describe the results of the testing for mycoplasma contamination OR declare that the cell lines were not tested for mycoplasma contamination.*
Commonly misidentified lines<br>(See ICLAC register)	*Name any commonly misidentified cell lines used in the study and provide a rationale for their use.*

Palaeontology and Archaeology

Specimen provenance	*Provide provenance information for specimens and describe permits that were obtained for the work (including the name of the issuing authority, the date of issue, and any identifying information). Permits should encompass collection and, where applicable, export.*
Specimen deposition	*Indicate where the specimens have been deposited to permit free access by other researchers.*
Dating methods	*If new dates are provided, describe how they were obtained (e.g. collection, storage, sample pretreatment and measurement), where they were obtained (i.e. lab name), the calibration program and the protocol for quality assurance OR state that no new dates are provided.*

☐ Tick this box to confirm that the raw and calibrated dates are available in the paper or in Supplementary Information.

Ethics oversight	*Identify the organization(s) that approved or provided guidance on the study protocol, OR state that no ethical approval or guidance was required and explain why not.*

Note that full information on the approval of the study protocol must also be provided in the manuscript.

Animals and other research organisms

Policy information about studies involving animals; ARRIVE guidelines recommended for reporting animal research, and Sex and Gender in Research

Laboratory animals	*For laboratory animals, report species, strain and age OR state that the study did not involve laboratory animals.*
Wild animals	*Provide details on animals observed in or captured in the field; report species and age where possible. Describe how animals were*

Wild animals | caught and transported and what happened to captive animals after the study (if killed, explain why and describe method; if released, say where and when) OR state that the study did not involve wild animals.

Reporting on sex | Indicate if findings apply to only one sex; describe whether sex was considered in study design, methods used for assigning sex. Provide data disaggregated for sex where this information has been collected in the source data as appropriate; provide overall numbers in this Reporting Summary. Please state if this information has not been collected. Report sex-based analyses where performed, justify reasons for lack of sex-based analysis.

Field-collected samples | For laboratory work with field-collected samples, describe all relevant parameters such as housing, maintenance, temperature, photoperiod and end-of-experiment protocol OR state that the study did not involve samples collected from the field.

Ethics oversight | Identify the organization(s) that approved or provided guidance on the study protocol, OR state that no ethical approval or guidance was required and explain why not.

Note that full information on the approval of the study protocol must also be provided in the manuscript.

Clinical data

Policy information about clinical studies
All manuscripts should comply with the ICMJE guidelines for publication of clinical research and a completed CONSORT checklist must be included with all submissions.

Clinical trial registration | NCT04297436

Study protocol | Available in Supplementary Information

Data collection | Cohort participants 18-25 years were screened for eligibility and excluded from the study when they were pregnant, known to have severe allergic reactions, or bleeding disorders, or when living with HIV their viral load was confirmed to be >= 200 copies per mL. The trial was conducted between June 2021 and February 2022 using Bexsero®, a licensed Nm vaccine, in 50 participants aged 18 to 25 years who attended the Kenya Medical Research Institute (KEMRI) clinics in Mtwapa and Malindi, Kenya.

Outcomes | Gonorrhea was diagnosed in men with urethral or rectal discharge, in men who reported receptive anal intercourse, and in women irrespective of symptoms. Samples were obtained by swabbing and screening for N. gonorrhoeae by Gram stain, the oxidase test, and API-NH (bioMerieux, France). Routinely, volunteers who reported rectal anal intercourse (RAI) were offered proctoscopy. Since 2016, participants who reported receptive anal intercourse had a swap collected for GeneXpert® CT/NG assay (Cepheid AB, Sweden).

Dual use research of concern

Policy information about dual use research of concern

Hazards

Could the accidental, deliberate or reckless misuse of agents or technologies generated in the work, or the application of information presented in the manuscript, pose a threat to:

No	Yes	
☒	☐	Public health
☒	☐	National security
☒	☐	Crops and/or livestock
☒	☐	Ecosystems
☒	☐	Any other significant area

Experiments of concern

Does the work involve any of these experiments of concern:

No	Yes	
☒	☐	Demonstrate how to render a vaccine ineffective
☒	☐	Confer resistance to therapeutically useful antibiotics or antiviral agents
☒	☐	Enhance the virulence of a pathogen or render a nonpathogen virulent
☒	☐	Increase transmissibility of a pathogen
☒	☐	Alter the host range of a pathogen
☒	☐	Enable evasion of diagnostic/detection modalities
☒	☐	Enable the weaponization of a biological agent or toxin
☒	☐	Any other potentially harmful combination of experiments and agents

Plants

Seed stocks	*Report on the source of all seed stocks or other plant material used. If applicable, state the seed stock centre and catalogue number. If plant specimens were collected from the field, describe the collection location, date and sampling procedures.*
Novel plant genotypes	*Describe the methods by which all novel plant genotypes were produced. This includes those generated by transgenic approaches, gene editing, chemical/radiation-based mutagenesis and hybridization. For transgenic lines, describe the transformation method, the number of independent lines analyzed and the generation upon which experiments were performed. For gene-edited lines, describe the editor used, the endogenous sequence targeted for editing, the targeting guide RNA sequence (if applicable) and how the editor was applied.*
Authentication	*Describe any authentication procedures for each seed stock used or novel genotype generated. Describe any experiments used to assess the effect of a mutation and, where applicable, how potential secondary effects (e.g. second site T-DNA insertions, mosiacism, off-target gene editing) were examined.*
